# Chemically driven design of N-doped MXene quantum dots for portable sensing and smartphone-integrated platforms

**DOI:** 10.1039/d6ra00830e

**Published:** 2026-04-30

**Authors:** Ghada Al-Assi, Abbas Hashim Abdulsalam, Roopashree R, Subhashree Ray, Baraa Mohammed Yaseen, Kavitha V, Renu Sharma, Aashna Sinha, Shaima Messa

**Affiliations:** a Faculty of Allied Medical Sciences, Hourani Center for Applied Scientific Research, Al-Ahliyya Amman University Amman Jordan; b College of Health and Medical Technologies, Department of Medical Laboratory Techniques, AL-Turath University Baghdad Iraq; c Department of Chemistry and Biochemistry, School of Sciences, JAIN (Deemed to be University) Bangalore Karnataka India; d Department of Biochemistry, IMS and SUM Hospital, Siksha 'O' Anusandhan (Deemed to be University) Bhubaneswar Odisha-751003 India; e Department of Medical Laboratory Technics, College of Health and Medical Technology, Alnoor University Mosul Iraq; f Department of Chemistry, Sathyabama Institute of Science and Technology Chennai Tamil Nadu India; g Department of Chemistry, University Institute of Sciences, Chandigarh University Mohali Punjab India; h School of Applied and Life Sciences, Division of Research and Innovation, Uttaranchal University Dehradun Uttarakhand India; i Young Researchers and Elite Club, Tehran Branch, Islamic Azad University Tehran Iran messashaimaa@gmail.com

## Abstract

Nitrogen-doped MXene quantum dots (N-MQDs) have recently emerged as versatile nanomaterials for portable sensing owing to their tunable surface chemistry, defect-rich structure, and favorable optical and electrochemical properties. This review presents a chemically driven perspective on the design of N-MQDs, emphasizing how controlled nitrogen incorporation, defect engineering, and surface termination modulation govern their functional behavior in miniaturized sensing systems. Rather than focusing solely on analytical performance, the discussion highlights material-level design principles that enable stable integration of N-MQDs into portable and smartphone-integrated platforms. Key strategies for physical anchoring, spatial organization, optical coupling, and mechanical robustness are critically examined to clarify how nanoscale chemical features translate into reliable platform-level performance. Representative examples of fluorescence-based, electrochemical, and dual-mode sensing architectures are summarized to illustrate the adaptability of N-MQDs across environmental and bioanalytical applications. By connecting chemical design with architectural integration, this review provides a unified framework for developing next-generation MQD-based sensing platforms compatible with decentralized, user-friendly, and smartphone-assisted diagnostics.

## Abstract 2

N-doped MXene quantum dots enable portable sensing by linking nitrogen doping, defect engineering, and surface chemistry with fluorescence, electrochemical, and smartphone-based platforms for environmental and bioanalytical detection.

## Graphical abstract

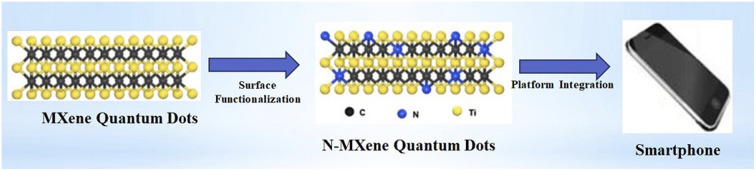

## 1. Introduction

The rapid expansion of portable and decentralized sensing technologies has reshaped the landscape of chemical and bioanalytical detection.^1,2^ Increasing demands for on-site environmental monitoring, resource-limited diagnostics, and real-time data acquisition have driven the development of sensing platforms that are compact, user-friendly, and compatible with consumer electronics.^3,4^ In this context, nanostructured materials play a pivotal role by enabling signal transduction mechanisms that remain effective at reduced device scales.^5,6^ Among emerging nanomaterials, MXene-based quantum dots have attracted growing interest due to their unique combination of surface tunability, electronic conductivity, and compatibility with miniaturized architectures.^7,8^

MXenes, a family of two-dimensional transition metal carbides and nitrides, exhibit rich surface chemistry arising from abundant functional terminations and defect sites.^9^ When processed into quantum-confined domains, MXene quantum dots (MQDs) inherit these features while gaining size-dependent optical and electronic characteristics.^10,11^ The high surface-to-volume ratio and defect density of MQDs make them particularly responsive to chemical modification, positioning them as adaptable building blocks for portable sensing applications. However, the functional performance of these materials is not intrinsic but is strongly governed by how their chemical structure is engineered at the nanoscale.^12–14^

Nitrogen doping has emerged as an effective strategy to modulate the properties of MQDs. The incorporation of nitrogen atoms into defect sites, surface regions, or carbon sublattices alters charge distribution, electronic states, and surface reactivity without requiring complex post-processing.^15,16^ Importantly, nitrogen doping is not a singular modification but a chemically driven design process influenced by synthesis pathways, defect landscapes, and surface terminations. These factors collectively determine how N-MQDs interact with their surrounding environment and how they respond to external stimuli in sensing configurations. As such, understanding nitrogen doping from a chemical design perspective is essential for rational material optimization.^17,18^

While a growing body of literature reports the analytical performance of N-MQDs in detecting ions, small molecules, and biomolecules, less attention has been paid to their integration into practical sensing platforms.^19,20^ In portable and smartphone-integrated systems, material performance cannot be decoupled from physical architecture. Parameters such as anchoring stability, spatial distribution, optical coupling, and mechanical resilience critically influence signal reliability, reproducibility, and user-independent operation. Without deliberate platform-level integration, even chemically optimized quantum dots may fail to deliver consistent performance outside laboratory conditions.^21–24^

Smartphone-based sensing platforms represent a particularly impactful direction for decentralized analysis. By leveraging built-in cameras, illumination sources, and data processing capabilities, smartphones offer an accessible interface for translating nanoscale material responses into quantifiable outputs.^25–28^ For MQD-based sensors, compatibility with smartphone readouts imposes additional constraints on film uniformity, optical path control, and signal stability. These requirements highlight the necessity of aligning chemical design with architectural considerations to ensure that material functionality is preserved during system-level integration.^29–31^

Existing reviews on MQDs have predominantly focused on synthesis routes, photophysical properties, or specific sensing mechanisms.^15–17,19,32,33^ However, a comprehensive framework connecting chemically driven material design with portable and smartphone-integrated sensing platforms remains underdeveloped. Addressing this gap is critical for advancing MQDs from proof-of-concept materials to reliable components in real-world sensing technologies.

This review aims to bridge chemical design principles and platform-level integration strategies for nitrogen-doped MXene quantum dots (N-MQDs) in portable sensing applications. Emphasis is placed on how nitrogen incorporation, defect engineering, and surface chemistry influence material behavior, and how these features enable stable integration into miniaturized and smartphone-compatible platforms. By shifting the focus from isolated sensing performance to material–platform coherence, this work provides a structured perspective to guide the development of next-generation MQD-based sensing systems suitable for decentralized and user-oriented applications.

## 2. nitrogen coordination chemistry and defect-driven doping strategies in MQDs

### 2.1. Overview of MQDs: structure and physicochemical properties

MQDs represent the zero-dimensional derivatives of two-dimensional MXenes, typically obtained through top-down exfoliation and fragmentation of layered transition-metal carbides, nitrides, or carbonitrides with a general formula of M_*n*+1_X_*n*_T_*x*_ (where M is an early transition metal, X represents carbon and/or nitrogen, and T_*x*_ denotes surface terminations such as –O, –OH, and –F). Compared with their parent MXene nanosheets, MQDs possess lateral dimensions typically below 10 nm, which results in pronounced quantum confinement and abundant edge sites. These structural characteristics lead to unique physicochemical properties that are particularly advantageous for sensing applications.^10,11^

The small size and large surface-to-volume ratio of MQDs significantly increase the density of active sites available for molecular adsorption and surface reactions. In addition, the presence of rich surface functional groups derived from MXene terminations facilitates strong interactions with various analytes through electrostatic interactions, hydrogen bonding, and coordination effects. Such surface chemistry also enables straightforward functionalization and heteroatom doping, which can further tailor their electronic structure.

From an electronic perspective, MQDs inherit the high electrical conductivity and metallic or semi-metallic nature of MXenes while simultaneously exhibiting size-dependent electronic states induced by quantum confinement. These effects often result in tunable band structures and enhanced charge-transfer processes, which are critical for electrochemical and optical sensing mechanisms. Furthermore, MQDs frequently display strong and stable photoluminescence behavior due to surface states and defect-related emission, making them promising candidates for fluorescence-based sensing platforms.^12,13^

The combination of high conductivity, tunable surface chemistry, abundant defect sites, and strong photoluminescent responses makes MQDs highly attractive nanomaterials for sensing technologies. These features enable efficient signal transduction, improved sensitivity, and selective interactions with target analytes, thereby supporting their rapidly expanding use in chemical and biological sensing systems.

### 2.2. Synthetic strategies for nitrogen-doped MXene quantum dots

N-MQDs are generally produced through chemical strategies that simultaneously induce the fragmentation of two-dimensional MXene nanosheets and enable the incorporation of nitrogen heteroatoms into the emerging nanostructure. Among the various approaches reported in the literature, hydrothermal and solvothermal synthesis represent the most widely employed routes due to their simplicity, scalability, and ability to provide controlled reaction environments for both size reduction and heteroatom incorporation. In these processes, multilayer or delaminated MXene precursors, such as Ti_3_C_2_T_*x*_, are dispersed in aqueous or organic solvents containing nitrogen-rich compounds including ammonia, urea, ethylenediamine, or other amine-based molecules that function as nitrogen donors.^17–20^

Under elevated temperature and autogenous pressure, the MXene sheets undergo chemical fragmentation through oxidative cutting or hydrolytic processes, producing nanoscale fragments that evolve into quantum dots typically smaller than 10 nm. Simultaneously, nitrogen-containing precursors decompose or react with surface functional groups of MXene, generating reactive nitrogen species capable of interacting with exposed metal or carbon sites. These reactions enable the incorporation of nitrogen atoms during the formation of the quantum dots, rather than through a separate post-doping step, which improves dopant dispersion and structural stability.

Reaction parameters strongly influence the characteristics of the resulting N-MQDs. Higher reaction temperatures and longer durations generally accelerate the fragmentation of MXene sheets and increase the probability of nitrogen incorporation, while the concentration and chemical nature of the nitrogen precursor determine the overall doping level.^23–26^ Solvothermal systems based on organic amines can also act as surface-passivating agents, helping stabilize the ultrasmall nanoparticles and prevent aggregation during synthesis.

**Fig. 1:**
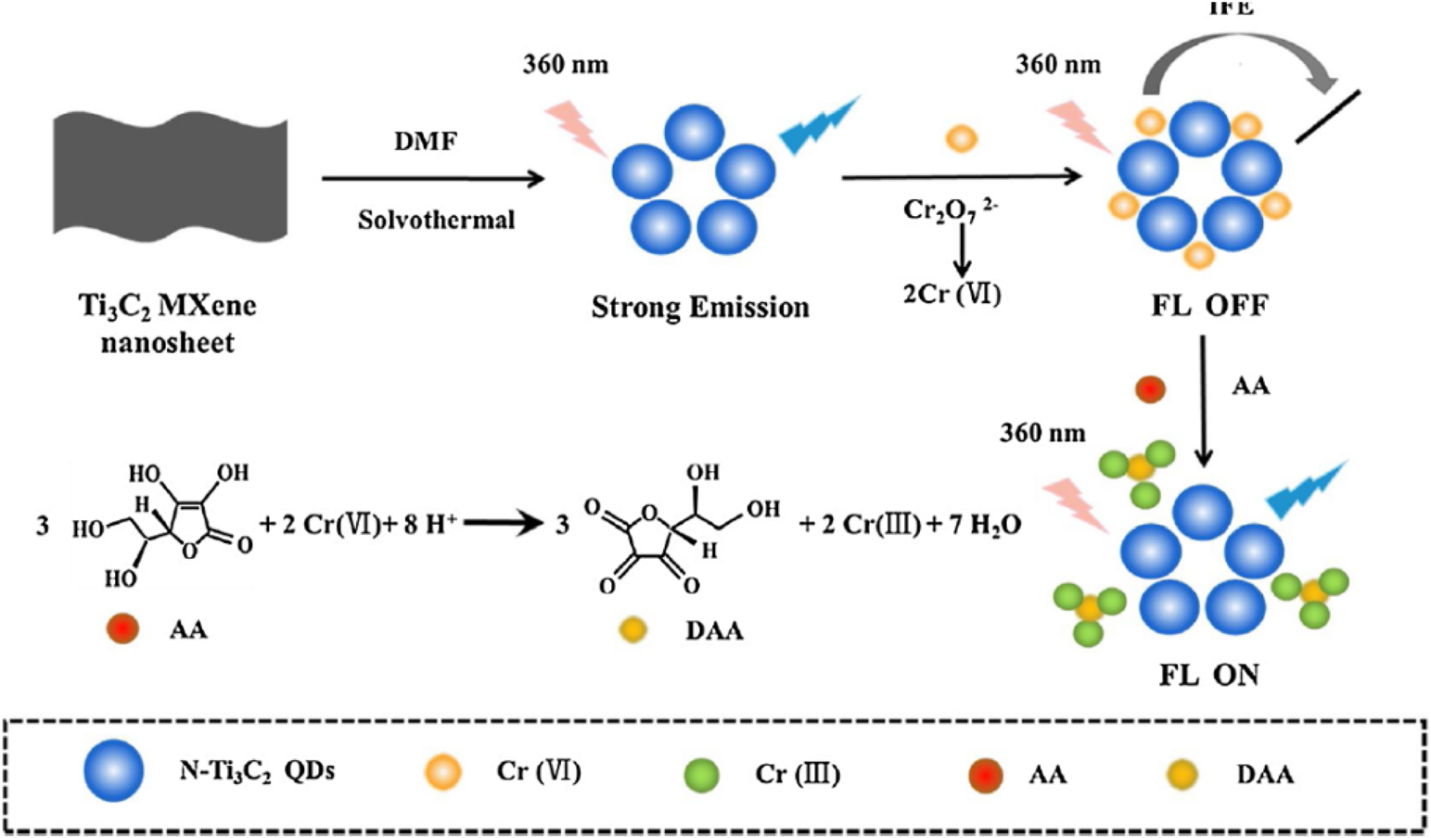
Schematic of the one-pot solvothermal synthesis of Ti_3_C_2_ MQDs from MXene nanosheets using N-rich precursors. Adapted with permission from ref. 33. © 2021 Elsevier B.V.

Fig. 1 illustrates the general synthetic pathway for N-MQDs. Initially, exfoliated MXene nanosheets serve as the structural precursor. During hydrothermal or solvothermal treatment, these sheets undergo controlled chemical cutting, generating nanoscale fragments that evolve into quantum dots. Concurrently, nitrogen-containing molecules decompose or react with surface sites, enabling nitrogen atoms to become incorporated into the developing nanostructure. This integrated synthesis mechanism allows simultaneous control of particle size, surface chemistry, and dopant incorporation, providing a versatile platform for preparing N-MQDs with tunable structural and chemical characteristics.

### 2.3. Structural evolution during nitrogen incorporation in MQDs

The incorporation of nitrogen into MXene quantum dots induces significant structural evolution within the nanoscale lattice, influencing both local atomic arrangements and the overall stability of the nanostructure. During hydrothermal or solvothermal synthesis, the fragmentation of two-dimensional MXene sheets produces ultrasmall nanodomains characterized by abundant edge sites, lattice imperfections, and under-coordinated transition-metal atoms. These highly reactive regions serve as preferential anchoring sites for nitrogen-containing species generated from precursor decomposition, facilitating the stabilization of nitrogen atoms within the forming quantum-dot framework.

Because MQDs possess dimensions typically below 10 nm, their atomic structure is dominated by surface and edge atoms rather than bulk lattice sites.^30–34^ This high surface-to-volume ratio significantly enhances the chemical reactivity of the nanomaterial and increases the density of available coordination environments for heteroatom incorporation. As nitrogen species interact with the fragmented MXene lattice, they can occupy energetically favorable sites near defects, vacancies, or unsaturated metal centers. The resulting interactions often lead to localized lattice distortions, redistribution of electronic charge, and the formation of new bonding environments that stabilize the doped structure.

Another important factor governing nitrogen incorporation is the coupling between lattice reconstruction and defect generation during the quantum-dot formation process. As MXene sheets are chemically cleaved into nanoscale fragments, numerous structural defects—including carbon vacancies, metal vacancies, and edge terminations—emerge within the lattice. These defects provide energetically favorable sites for nitrogen accommodation, thereby facilitating the integration of nitrogen species into the evolving nanostructure. In many cases, the presence of nitrogen helps stabilize these defect sites by saturating dangling bonds and reducing structural instability at the nanoscale.^35–37^ The incorporation of nitrogen not only modifies the atomic arrangement of MQDs but also alters the local electronic environment surrounding the doped regions. Such structural and electronic modifications establish the foundation for the diverse physicochemical properties of N-MQDs discussed in the following sections, including specific nitrogen bonding configurations, defect-mediated interactions, and surface chemical effects that collectively govern their functional performance.

### 2.4. Nitrogen bonding configurations and local coordination states

Nitrogen atoms incorporated into MQDs can adopt multiple bonding configurations, each associated with distinct local coordination environments and electronic consequences. Commonly identified nitrogen states include pyridinic-like, pyrrolic-like, graphitic (quaternary), and metal-coordinated nitrogen species. In carbide-based MXene QDs, metal–nitrogen coordination plays a particularly prominent role due to the strong affinity between early transition metals (*e.g.*, Ti, V, Nb) and nitrogen donors.^38^ Pyridinic and pyrrolic nitrogen species are typically localized at edge sites or defect-rich regions, where incomplete lattice coordination allows nitrogen to stabilize unsaturated carbon or metal atoms. These configurations introduce localized lone-pair electrons and modify the electron density distribution near the Fermi level. In contrast, graphitic nitrogen involves substitution within the carbon sublattice, resulting in a more delocalized electronic contribution and minimal geometric distortion. Metal–nitrogen coordination, often overlooked in simplified models, can significantly alter the local crystal field and influence metal d-orbital occupancy.^39^

The coexistence of multiple nitrogen coordination states is not merely a structural artifact but a defining chemical feature of N-MXene QDs. Their relative abundance depends on synthesis conditions and directly governs interfacial chemical reactivity. Advanced spectroscopic analyses consistently reveal that these nitrogen species do not act independently; rather, they form chemically coupled domains that collectively shape the local coordination landscape, making nitrogen doping a multidimensional chemical modification rather than a single-variable adjustment.^38,40^

**Fig. 2:**
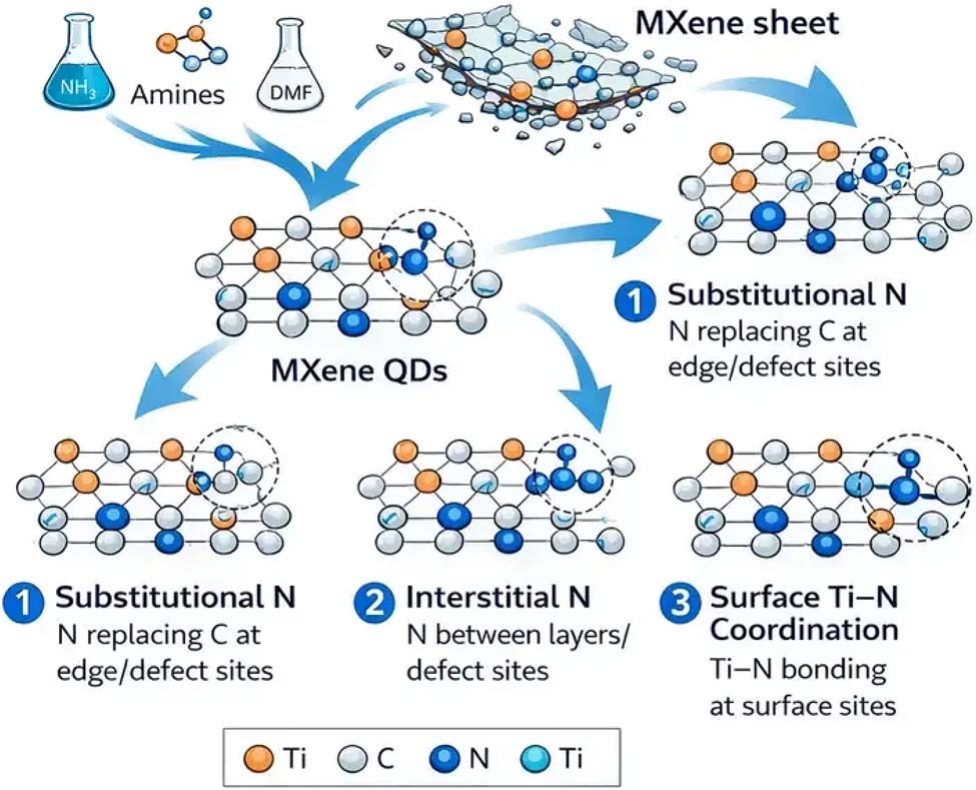
Schematic representation of nitrogen bonding configurations and defect–dopant coupling in MQDs, showing pyridinic, pyrrolic, graphitic, and Ti–N coordination as well as nitrogen incorporation at carbon vacancy sites and the resulting charge redistribution within the MQD lattice.

The below figure illustrates the atomic configurations of nitrogen incorporation and defect–dopant coupling mechanisms in MXene quantum dots. Different nitrogen bonding environments are depicted within the MQD lattice, including pyridinic N, pyrrolic N, graphitic N, and Ti–N coordination, each representing distinct electronic interactions with the surrounding Ti–C framework. Pyridinic and pyrrolic nitrogen are typically located at edge or defect sites, while graphitic nitrogen substitutes carbon atoms within the lattice plane. In addition, the schematic demonstrates how carbon vacancies act as favorable sites for nitrogen incorporation. When nitrogen occupies these vacancies, local charge redistribution occurs, modifying the electronic structure and stabilizing the doped configuration. These defect–dopant interactions play a key role in tuning the electronic, catalytic, and sensing properties of nitrogen-doped MXene quantum dots (Fig. 2).

**Fig. 3:**
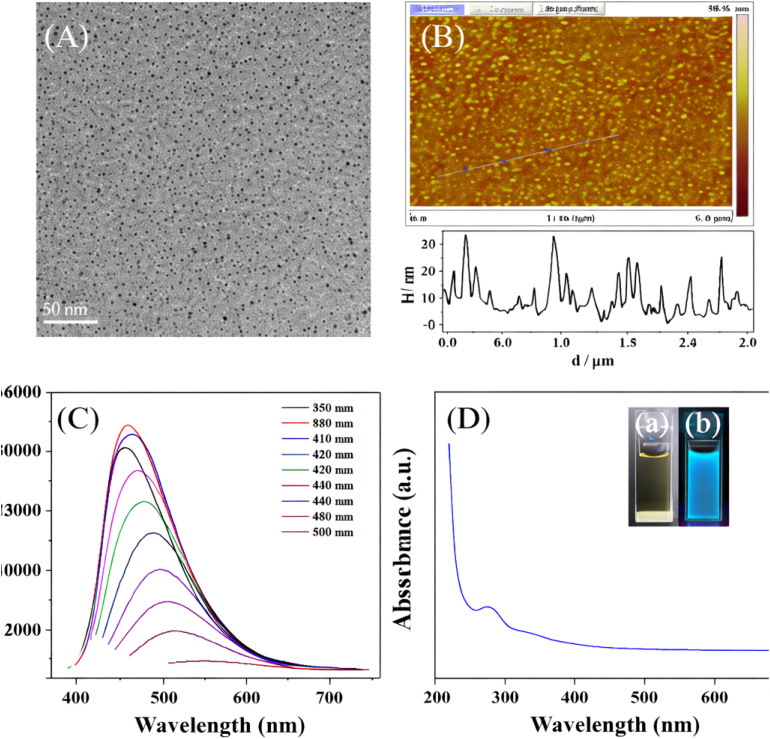
Characterization of N-doped Ti_3_C_2_ quantum dots. (A) TEM image showing uniform particle sizes of 2–5 nm. (B) AFM image confirming few-layer structure with heights of 1–5 nm. (C) Excitation-dependent fluorescence spectra with maximum excitation at 395 nm and emission at 458 nm. (D) UV–vis absorption spectrum (peak at ∼273 nm) with insets showing pale brown color under visible light (a) and bright blue fluorescence under 365 nm UV light (b). Adapted with permission from ref. 71. © 2022 Elsevier B.V.

The panels of Fig. 3(A–D) provide direct characterization of the structural and optical features of N-doped Ti_3_C_2_ QDs, offering valuable experimental evidence for understanding nitrogen bonding configurations and local coordination states, in close alignment. Panels A (TEM image) and B (AFM image) establish the structural foundation that enables diverse nitrogen coordination environments in N-doped Ti_3_C_2_ QDs. The uniform particle size distribution in the 2–5 nm range (TEM) and topographic heights of 1–5 nm (AFM) confirm a highly exfoliated, few-layer structure with quantum-confined dimensions. This nanoscale confinement dramatically increases the proportion of edge sites, defect regions, and under-coordinated Ti centers relative to bulk MXene counterparts, creating chemically reactive loci that favor heterogeneous nitrogen incorporation. Such structural features directly facilitate the formation of multiple nitrogen bonding motifs—particularly pyridinic-like and pyrrolic-like configurations at edges and defects, as well as potential metal–nitrogen coordination at exposed transition-metal sites—by lowering kinetic barriers and stabilizing otherwise strained dopant geometries.

Panel C (fluorescence excitation-emission spectra) reveals excitation-dependent photoluminescence behavior that reflects the electronic consequences of varied nitrogen coordination states. The progressive red-shift of emission peaks with increasing excitation wavelength (380–500 nm), culminating in maximum excitation at 395 nm and emission at 458 nm, indicates a distribution of emissive trap states arising from distinct local coordination environments. Pyridinic and pyrrolic nitrogen species, typically localized at edge or defect sites, introduce lone-pair electrons and mid-gap states that broaden the density of available radiative pathways, while metal-coordinated nitrogen can further modulate d-orbital hybridization and crystal-field splitting at Ti centers. This multi-configurational nitrogen landscape—rather than a single uniform dopant type—produces the observed polydisperse emissive centers, underscoring the coexistence of chemically coupled nitrogen domains that collectively govern optical properties.

Panel D (UV–vis absorption spectrum with photographic insets) complements the fluorescence data by highlighting surface-state contributions linked to nitrogen-induced electronic perturbations. The characteristic absorption peak near 273 nm, combined with vivid blue luminescence under 365 nm irradiation (*versus* pale brown under visible light), arises from quantum confinement and surface functionalization effects amplified by nitrogen doping. The bright blue emission originates from isolated sp^2^ domains within the carbon–oxygen matrix, where nitrogen incorporation—through pyridinic, pyrrolic, or amino configurations—modifies local electronegativity and orbital hybridization, generating bandgap states consistent with the observed emission energy. Collectively, these optical signatures demonstrate how diverse nitrogen bonding configurations and their associated local coordination environments engineer a rich manifold of electronic states, distinguishing N-doped Ti_3_C_2_ QDs from undoped analogs and enabling tunable optoelectronic functionality.

### 2.5. Defect chemistry and nitrogen–vacancy interactions in quantum-confined MXenes

Defect chemistry plays a central role in stabilizing nitrogen dopants within MQDs. The formation of carbon vacancies, metal vacancies, and mixed vacancy complexes during exfoliation and size reduction creates chemically active sites that strongly interact with nitrogen species. In the quantum dot regime, the high defect density is not a limitation but a chemical advantage, providing anchoring points that lower the formation energy of nitrogen-doped configurations.^34,41^

Nitrogen–vacancy interactions often lead to the formation of stable defect complexes, where nitrogen atoms passivate dangling bonds or reconstruct local coordination geometries. Carbon vacancies, in particular, serve as energetically favorable sites for substitutional nitrogen, enabling strong covalent bonding with adjacent metal atoms. These interactions reduce lattice strain while simultaneously altering the local electronic environment, resulting in defect-stabilized doping motifs that are rarely achievable in extended MXene sheets. Furthermore, the spatial confinement inherent to quantum dots amplifies defect–dopant coupling effects. Localized lattice distortions and charge redistribution extend across the entire nanodomain, effectively transforming point defects into global chemical modifiers.^42,43^ This phenomenon underscores the necessity of treating defect chemistry and nitrogen doping as inseparable processes in N-MXene QDs, where dopant behavior cannot be decoupled from the underlying defect topology.

### 2.6. Influence of surface terminations on nitrogen coordination chemistry

Surface terminations intrinsic to MXenes exert a profound influence on nitrogen coordination chemistry in quantum dots. Functional groups such as –O, –OH, and –F define the chemical accessibility of surface metal sites and regulate the adsorption, activation, and incorporation of nitrogen-containing species. In N-MXene QDs, the high surface-to-volume ratio magnifies termination effects, making surface chemistry a dominant factor in dopant stabilization. Oxygen-terminated surfaces tend to promote stronger metal–nitrogen interactions by facilitating partial charge transfer and coordination bond formation.^44–46^ Hydroxyl groups, on the other hand, introduce hydrogen-bonding networks that can transiently stabilize nitrogen precursors during synthesis, influencing dopant distribution. Fluorine terminations generally reduce nitrogen incorporation efficiency due to their strong metal–fluorine bonds, which limit available coordination sites.

Crucially, nitrogen doping can also induce termination rearrangement, leading to dynamic surface reconstruction. This bidirectional interaction between dopants and surface terminations results in chemically heterogeneous surfaces, where local coordination environments differ markedly across a single quantum dot. Such complexity challenges simplified structural models but provides a chemically rich platform for tailoring interfacial properties through controlled termination engineering.^35,47^

**Fig. 4:**
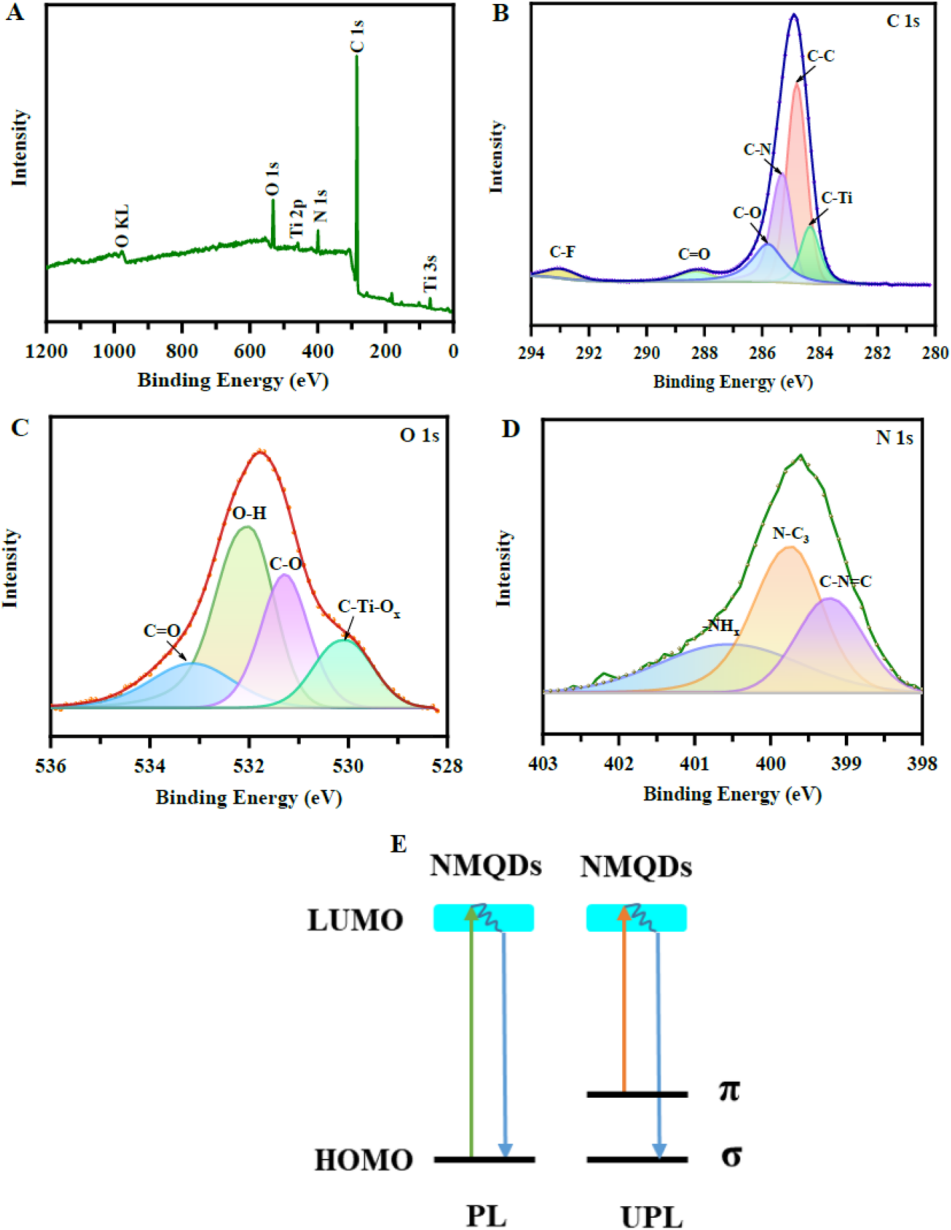
XPS analysis of nitrogen-doped Ti_3_C_2_T_*x*_ MXene quantum dots showing heterogeneous surface terminations and nitrogen coordination. (A) Survey spectrum confirming Ti, C, O, N, and F. (B) C 1s deconvolution revealing C–Ti, C–C/C=C, C–O, C–F, and C–N bonds. (C) O 1s components corresponding to O–H, C–O, C=O, and C–Ti–O_*x*_ species. (D) N 1s spectrum indicating pyridinic, pyrrolic, and amine-type nitrogen configurations. (E) Schematic illustration of down-conversion and up-conversion photoluminescence enabled by nitrogen-modified surface states. Adapted with permission from ref. 37. © 2023 American Chemical Society.

Fig. 4 systematically correlates the XPS fingerprints of N-doped Ti_3_C_2_T_*x*_ MXene quantum dots with the role of surface terminations in governing nitrogen coordination chemistry. Panel (A) confirms the coexistence of Ti, C, O, N, and residual F, directly evidencing that nitrogen incorporation occurs within a termination-rich surface environment rather than an idealized carbide lattice. As discussed in the text, intrinsic –O, –OH, and –F terminations inherited from the etching process dictate the chemical accessibility of surface Ti sites. Oxygen-terminated and hydroxylated regions provide favorable coordination environments that stabilize nitrogen species through partial charge transfer and hydrogen bonding, whereas fluorinated domains remain comparatively inert, limiting local nitrogen uptake. This uneven distribution of terminations establishes a chemically heterogeneous surface, which is a defining characteristic of N-MXene QDs and a prerequisite for programmable interfacial behavior.

Panels (B) and (C) further resolve how this termination landscape evolves upon nitrogen doping. The C 1s spectrum reveals contributions from C–Ti, C–C/C=C, C–O, C–F, and C–N bonds, while the O 1s spectrum identifies O–H, C–O, C=O, and C–Ti–O_*x*_ species. These features directly support the notion that nitrogen incorporation does not occur in isolation but is coupled to termination rearrangement and surface reconstruction. Oxygen-rich terminations promote stronger metal–nitrogen coordination by activating Ti sites and facilitating dopant stabilization, in agreement with prior reports cited in the text. Simultaneously, hydroxyl groups can transiently stabilize nitrogen-containing intermediates during synthesis, influencing dopant distribution, while fluorine terminations persist as coordination-limiting domains. The resulting surface is therefore not uniformly doped but composed of locally distinct coordination environments across individual quantum dots.

The N 1s spectrum in panel (D) provides direct spectroscopic evidence for this coordination diversity, showing pyridinic N–C_3_, pyrrolic C–N=C, and –NH_2_/–NH species. These configurations reflect substitutional, edge-associated, and surface-grafted nitrogen states that emerge from the bidirectional interaction between dopants and surface terminations. Such chemically heterogeneous nitrogen coordination generates multiple electronic states, which are schematically summarized in panel (E). Here, nitrogen-modified surface states enable both down-conversion (π* → π) and up-conversion (σ → π*) photoluminescence pathways, illustrating how termination-driven nitrogen chemistry directly translates into tunable optoelectronic behavior. Collectively, Fig. 4 reinforces the central argument that surface terminations are not passive spectators but active regulators of nitrogen coordination, surface reconstruction, and functional signal routing in N-MXene quantum dots.

### 2.7. Electronic structure modulation arising from nitrogen coordination chemistry

Nitrogen coordination chemistry in MQDs directly modulates their electronic structure through both local and collective effects. At the atomic scale, nitrogen dopants alter charge density distribution, introduce mid-gap or near-Fermi-level states, and modify metal–carbon hybridization. These changes are highly sensitive to the specific coordination environment of nitrogen, with metal-coordinated nitrogen producing markedly different electronic perturbations compared to carbon-substituted configurations.^48^

In quantum-confined systems, electronic structure modulation extends beyond localized states. Due to the small domain size, dopant-induced electronic perturbations can influence the entire density of states profile, effectively redefining the electronic identity of the quantum dot. This global sensitivity distinguishes N-MXene QDs from their bulk counterparts and enables fine-tuning of electronic properties through precise control of nitrogen chemistry. Importantly, this modulation arises from chemical coordination effects rather than extrinsic device architecture or external stimuli. As such, nitrogen doping serves as an intrinsic electronic design tool, embedded at the atomic level.^49–51^ Understanding and controlling this coordination-driven electronic modulation is essential for rationally linking chemical structure to higher-level functional performance, which will be addressed in subsequent sections without revisiting the chemical foundations established here.

The Table 1 consolidates key aspects of nitrogen incorporation in MQDs, linking doping pathways, nitrogen bonding motifs, defect interactions, surface termination effects, and resultant electronic structure modulation. Each row corresponds to a distinct doping feature or local coordination environment, while columns summarize mechanistic insights, structural consequences, electronic effects, functional implications, and synthetic strategies. This integrated presentation allows readers to rapidly assess how atomic-level variations in nitrogen chemistry influence quantum dot properties, including charge distribution, defect stabilization, and surface reactivity, while providing a practical guide for rationally designing N-MXene QDs for sensing, optoelectronic, or catalytic applications. By combining mechanistic, structural, and functional perspectives in a single framework, the table captures the multidimensional nature of doping chemistry without redundancy, serving as a concise reference for both experimental design and theoretical interpretation.

**Table 1:** Nitrogen doping chemistry and local coordination in MQDs: structural, electronic, and functional perspectives

Phenomenon/Feature	Mechanistic insight	Impact on local structure & electronic properties	Engineering/synthetic strategy	Ref.
Substitutional nitrogen incorporation	Nitrogen replaces carbon in carbide lattice or fills vacancies	Alters local hybridization, introduces mid-gap states	Controlled hydrothermal or solvothermal reactions, temperature tuning	33 and 34
Interstitial nitrogen incorporation	Nitrogen occupies lattice interstitial sites or defects	Minimal lattice distortion, modifies charge distribution	Plasma or thermal post-treatment for precise doping	35 and 36
Pyridinic/pyrrolic nitrogen	Edge or defect-localized N with lone pairs	Enhances local reactivity, provides coordination sites for analytes	Precursor selection and defect engineering	37 and 38
Graphitic (quaternary) nitrogen	Substitution within carbon sublattice	Delocalized electrons, stabilizes Fermi level, minimal strain	Optimized annealing conditions, solvent-mediated doping	39 and 40
Metal–nitrogen coordination	N binds to under-coordinated Ti/V/Nb atoms	Modifies d-orbital occupancy, tunes redox activity	Use of N-rich ligands during MXene exfoliation	41 and 42
Defect–dopant coupling	Vacancies anchor N atoms, forming stable complexes	Reduces lattice strain, stabilizes dopants, modifies electronic density	Controlled etching/exfoliation to introduce active defects	43 and 44
Surface termination interplay	–O, –OH, –F groups affect N incorporation	Surface heterogeneity, local coordination variation	Pre- or post-functionalization of MXene sheets	45 and 46
Dynamic surface reconstruction	Nitrogen induces termination rearrangement	Creates chemically heterogeneous surfaces, modifies adsorption behavior	Tuning precursor ratios and reaction kinetics	47 and 48
Charge redistribution	Dopant modifies electron density across QD	Alters optical and electrochemical behavior	Doping level optimization, co-doping strategies	49 and 50
Vacancy stabilization	Carbon/metal vacancies stabilize N dopants	Enhances fluorescence quantum yield and redox activity	Controlled defect density during top-down synthesis	51 and 52
Quantum confinement effects	Small QD size amplifies dopant influence	Global electronic modulation, enhanced sensitivity	Size-controlled exfoliation and QD isolation	36 and 41
Multi-site chemical coupling	N species form coupled domains	Cooperative effects on reactivity and electronic properties	Sequential doping, post-synthetic annealing, co-dopant design	43 and 48

## 3. Architectural and functional integration of N-MQDs in miniaturized and flexible sensing platforms

### 3.1. Interfacial anchoring of N-MQDs on solid substrates

The integration of N-MQDs into miniaturized sensing platforms begins with their stable anchoring onto solid substrates. At this stage, the primary challenge is not chemical reactivity but interfacial compatibility between the quantum dots and the supporting material. Substrate surfaces—ranging from cellulose-based matrices to polymeric and inorganic supports—present distinct physicochemical environments that dictate adhesion strength, spatial distribution, and long-term retention of the quantum dots.^52,53^

Anchoring mechanisms are governed by non-covalent interactions such as electrostatic attraction, hydrogen bonding, van der Waals forces, and coordination interactions with surface functionalities. The presence of surface terminations and defect sites on N-MXene QDs facilitates multivalent interactions, enabling uniform immobilization without the need for aggressive chemical grafting. This physical attachment preserves the intrinsic properties of the quantum dots while ensuring mechanical stability under handling and operational conditions.^35,54^

Crucially, anchoring strategies must balance adhesion strength with accessibility. Overly dense or encapsulating attachment can hinder mass transport and limit effective interaction with external chemical environments. Therefore, substrate coupling is optimized through controlled deposition techniques—such as drop-casting, inkjet printing, or layer-by-layer assembly—that enable reproducible coverage while maintaining nanoscale exposure.^55,56^ This interfacial anchoring step establishes the physical foundation upon which higher-level platform integration is constructed.

**Fig. 5:**
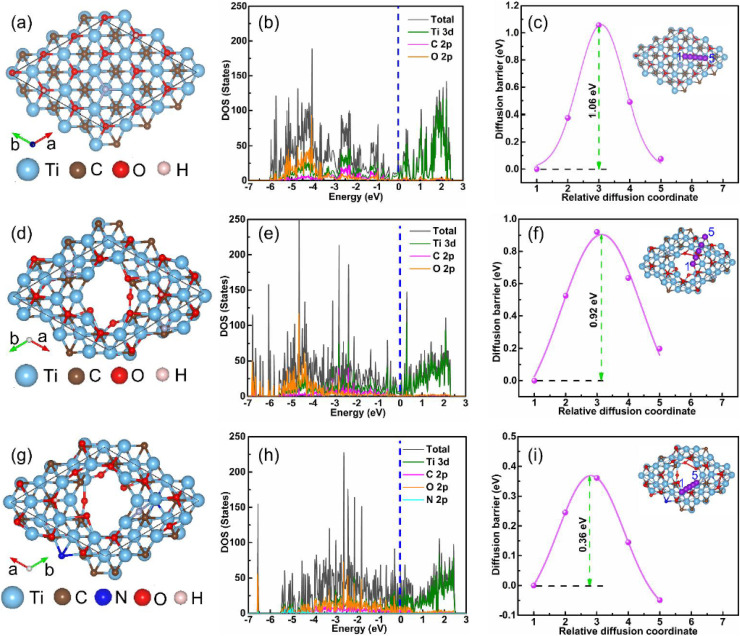
DFT-calculated atomic structures and electronic features of Ti_3_C_2_T_*x*_ nanosheets and MQDs highlighting nitrogen-induced surface electronic modulation relevant to interfacial anchoring. (a, d and g) Optimized geometries of O-terminated nanosheets, bare MQDs, and N-doped MQDs. (b, e and h) Total and partial density of states showing enhanced near-Fermi-level DOS and N 2p contributions in N-MQDs. (c, f and i) Calculated K^+^ diffusion barriers and migration pathways, reflecting increased surface electronic adaptability and carrier accessibility upon nitrogen doping. Adapted with permission from ref. 17. © 2024 Elsevier B.V.

Panels (a), (d), and (g) in Fig. 5 present the optimized atomic configurations of O-terminated Ti_3_C_2_T_*x*_ nanosheets, bare MQDs, and nitrogen-doped MQDs, respectively. The transformation from extended two-dimensional sheets to zero-dimensional quantum dots inherently increases the density of edge sites, undercoordinated Ti atoms, and surface terminations. Nitrogen incorporation further perturbs the local coordination environment by introducing heteroatom-centered motifs and redistributing surface charge density. These structural features are directly relevant to interfacial anchoring, as they define the availability and spatial distribution of interaction sites capable of engaging in electrostatic attraction, hydrogen bonding, van der Waals interactions, and weak coordination with functional groups present on solid substrates.

The electronic consequences of these structural modifications are captured in panels (b), (e), and (h), which display the total and partial density of states. In N-doped MQDs, the emergence of N 2p–derived states and the pronounced increase in DOS near the Fermi level indicate enhanced electronic polarizability and localized charge accumulation at the surface. From an interfacial perspective, this electronic enrichment strengthens non-covalent anchoring interactions by increasing surface dipole moments and facilitating charge-assisted adhesion to polar or functionalized substrates. Importantly, this mechanism enables robust immobilization without requiring covalent grafting or chemical modification of either the quantum dots or the substrate.

Panels (c), (f), and (i) further support this interpretation by demonstrating progressively reduced K^+^ diffusion barriers upon quantum confinement and nitrogen doping. Although presented as a transport metric, the lowered migration barriers reflect a softened and electronically adaptable surface potential landscape. Such surfaces are more accommodating to interfacial reorganization during deposition and drying processes, allowing N-MQDs to conform to substrate topography and maximize contact area. This adaptability enhances anchoring stability while preserving nanoscale exposure, a critical balance emphasized in controlled deposition strategies such as drop-casting and layer-by-layer assembly. Collectively, figure establishes a mechanistic bridge between atomic-scale electronic structure and macroscopic interfacial behavior. By demonstrating that nitrogen coordination intrinsically enhances surface electronic accessibility and energetic adaptability, the figure provides a rigorous theoretical foundation for the effective interfacial anchoring of N-MQDs on solid substrates.

### 3.2. Spatial organization and distribution control in miniaturized architectures

Beyond initial attachment, the spatial organization of N-MQDs within miniaturized architectures critically determines platform uniformity, reproducibility, and operational consistency. In contrast to continuous thin films, quantum dot assemblies inherently risk aggregation, clustering, and non-uniform coverage due to nanoscale interparticle interactions. Within compact sensing formats, such heterogeneities are amplified, as localized density variations directly influence macroscopic signal homogeneity and platform-to-platform variability.^57^

Spatial distribution control is primarily governed by physical deposition dynamics rather than chemical modification. Parameters such as solvent evaporation rate, wetting behavior, and surface energy gradients dictate lateral spreading and interparticle spacing during assembly. Techniques including capillary-driven self-organization, confined deposition within micro-patterned regions, and electrostatic alignment under weak external fields have been employed to guide quantum dot placement with high spatial fidelity. Importantly, these methods exploit kinetic and geometric constraints to regulate organization, ensuring that the chemical identity of N-MXene QDs remains unaltered during pattern formation.^58,59^

Long-term stability of the spatial arrangement represents an equally critical consideration. Quantum dot migration, aggregation, or detachment over time compromises signal reproducibility and undermines platform reliability. To mitigate these effects, miniaturized architectures incorporate kinetic trapping strategies such as nanoscale surface roughness, shallow confinement features, and porous substrates that restrict lateral mobility without inducing irreversible binding. These physical constraints effectively lock the desired spatial configuration in place while preserving accessibility.^60,61^ Through deliberate architectural design, spatial organization transforms dispersed quantum dots into a structurally coherent, device-compatible ensemble, establishing a robust physical foundation for miniaturized sensing platforms.

### 3.3. Integration into flexible and paper-based miniaturized supports

Flexible and paper-based substrates constitute a unique class of miniaturized supports characterized by mechanical compliance, intrinsic porosity, and structural heterogeneity. Integrating N-MQDs into such platforms requires strategies that accommodate deformation, capillary fluid transport, and repeated mechanical handling without compromising material retention or spatial integrity. Unlike rigid substrates, these supports demand coupling approaches that prioritize mechanical adaptability over permanent fixation.^62–64^

Integration within flexible matrices typically relies on physical entrapment rather than surface immobilization alone. The nanoscale dimensions of MQDs enable penetration into fiber networks or polymer meshes, where they adhere along internal surfaces and junctions. This volumetric distribution creates a three-dimensional embedding that distributes mechanical stress across the substrate, significantly reducing the risk of delamination or abrasion during bending, folding, or compression.^65^

A key challenge in such systems is maintaining functional accessibility while ensuring mechanical stability. Excessive infiltration into the bulk can limit exposure, whereas superficial deposition increases vulnerability to mechanical loss. Optimized integration therefore employs controlled infiltration depths, often combined with thin surface-stabilizing layers that protect embedded quantum dots without encapsulating them. These approaches ensure that N-MXene QDs remain both mechanically secured and physically accessible. Notably, all integration strategies in this context are defined by substrate mechanics and architecture, remaining fully independent of chemical sensing mechanisms or analyte-specific interactions.^34,66^

### 3.4. Coupling with miniaturized optical readout components

Miniaturized sensing platforms frequently incorporate compact optical readout elements, necessitating precise physical coupling between N-MQDs and light-interfacing components. At this level of integration, the primary considerations shift from substrate adhesion to optical path optimization, light confinement, and efficient signal collection within restricted geometries. These factors are governed by spatial alignment and architectural design rather than chemical modification.^67,68^

The relative positioning of quantum dots with respect to excitation sources and detectors defines the effective interaction volume and directly influences signal uniformity. Thin, homogeneous quantum dot layers reduce scattering losses and minimize reabsorption effects, while controlled thickness ensures consistent excitation penetration across the active area. Deposition techniques are therefore selected to achieve optical uniformity without altering intrinsic emission properties.

To further enhance signal extraction, miniaturized platforms often incorporate reflective backings, waveguiding structures, or optical isolation layers that suppress background interference. These components shape light propagation pathways and improve collection efficiency in confined formats. Importantly, the coupling between N-MXene QDs and optical readout elements is a purely geometrical and physical optimization problem.^69,70^ It functions independently of analyte interaction mechanisms and serves solely to translate nanoscale optical responses into macroscopically detectable signals compatible with compact device architectures.

### 3.5. Mechanical and environmental robustness in compact platform integration

Miniaturized sensing platforms are routinely subjected to mechanical stress, humidity fluctuations, and temperature variations during storage and operation. Ensuring that N-MQDs remain functionally integrated under these conditions requires platform-level stabilization strategies that address mechanical and environmental challenges without modifying the intrinsic properties of the quantum dots. Mechanical robustness is achieved through the use of flexible binders, compliant interlayers, and protective overlayers that absorb strain and mitigate shear forces. These elements distribute mechanical stress across the platform and prevent localized failure at the quantum dot–substrate interface.^71–73^ The nanoscale size of MQDs inherently reduces stress concentration, enabling conformal coverage even under deformation.

Environmental robustness further demands resistance to leaching, redistribution, and physical degradation. Platform designs incorporate microstructural confinement features, such as porous matrices or textured surfaces, which restrict quantum dot mobility while maintaining permeability. These strategies ensure long-term spatial stability without invoking chemical passivation or encapsulation.^36,74^ Critically, all robustness measures discussed here are platform-centric, preserving a strict conceptual boundary from chemical structure, coordination environments, or sensing function.

### 3.6. System-level compatibility with smartphone-integrated miniaturized platforms

At the system level, N-MQDs must be physically compatible with smartphone-integrated sensing configurations. This compatibility is defined by geometric alignment, form factor constraints, and consistency with consumer electronics hardware, rather than by sensing chemistry or analytical performance. Successful integration requires that quantum dot-based platforms operate as modular components within predefined device ecosystems. Platform geometries are engineered to align with smartphone camera modules, illumination sources, and accessory housings without requiring hardware modification. Thin-film formats, standardized dimensions, and reproducible mounting interfaces ensure consistent optical coupling and mechanical fit across devices. These constraints necessitate careful control over platform thickness, rigidity, and alignment features.^38,70^

Equally important is reproducibility across large numbers of units. System-level integration relies on defined mechanical tolerances and standardized assembly protocols to ensure user-independent operation. In this context, N-MXene QDs function as embedded material elements within a broader physical system.^41,75^ Their role is to remain structurally integrated and optically accessible, without encroaching upon application-specific evaluation or future design considerations addressed in subsequent sections.

## 4. Advanced analytical performance of N-MQDs in portable fluorescent and electrochemical sensing

### 4.1. Smartphone-integrated optical and electrochemical sensing platforms based on N-MQDs

The integration of nanomaterial-based sensors with smartphone-enabled analytical systems has emerged as a transformative approach for decentralized chemical and biological detection. In this context, N-MQDs offer a particularly suitable material platform due to their strong photoluminescence, tunable electronic structure, and high surface reactivity. These properties allow N-MQDs to function as efficient signal transducers whose optical or electrochemical responses can be readily captured and processed by smartphone hardware, enabling portable and user-friendly sensing systems.

In smartphone-integrated optical sensing configurations, the fluorescence emission of N-MQDs serves as the primary analytical signal. Upon interaction with target analytes, variations in photoluminescence intensity, wavelength, or color are generated through mechanisms such as fluorescence quenching, Förster resonance energy transfer (FRET), or charge-transfer interactions at the quantum-dot surface.^76,77^ These optical changes can be recorded using the smartphone camera, while dedicated mobile applications perform quantitative analysis through RGB color decomposition, intensity calibration, or image-based fluorescence mapping. Because modern smartphone cameras possess high pixel density and advanced image processing capabilities, they can function as compact optical detectors without requiring bulky spectroscopic equipment.

Beyond purely optical measurements, smartphone platforms can also interface with miniaturized electrochemical modules integrated into portable sensing devices. In such hybrid systems, N-MQDs deposited on screen-printed electrodes or conductive substrates generate analyte-dependent electrochemical signals that are transmitted to a smartphone through compact potentiostat interfaces. The smartphone subsequently performs signal acquisition, processing, and visualization, effectively transforming the mobile device into a multifunctional analytical workstation. This combination of fluorescence imaging and electrochemical readout enables dual-mode detection strategies that enhance analytical reliability through cross-validation of independent signal channels.

The practical implementation of smartphone-based sensing systems also requires careful consideration of optical alignment, illumination control, and signal normalization. Compact accessory modules are often employed to standardize the distance between the sensing substrate and the smartphone camera while incorporating controlled light sources such as LEDs to minimize environmental interference.^78–80^ In parallel, calibration algorithms and image-processing routines compensate for variations in ambient lighting and camera sensitivity, ensuring reproducible quantitative measurements across different devices.

Overall, the convergence of N-MQD nanomaterials with smartphone-assisted detection architectures provides a powerful framework for portable sensing technologies. By translating nanoscale photophysical and electrochemical responses into digital signals accessible through widely available consumer electronics, these systems enable rapid, low-cost, and field-deployable analytical platforms suitable for environmental monitoring, healthcare diagnostics, and on-site chemical analysis.

### 4.2. On/off/on fluorescent logic for selective arsenic detection in portable formats

N–Ti_3_C_2_ MQDs exhibiting bright yellow fluorescence represent a significant advancement in portable chemical sensing, especially for environmentally hazardous metal ions. While most MQDs emit in the blue region, the extended emission wavelength of 570 nm combined with a fluorescence quantum yield of 13.8% provides superior signal-to-noise ratios and deeper penetration in turbid media, which is particularly relevant for on-site water quality monitoring.^76^ This longer-wavelength fluorescence reduces interference from background fluorescence commonly observed in environmental samples, facilitating accurate detection under field conditions.

The sensing mechanism relies on well-defined “on/off/on” fluorescence logic. Initially, As^3+^ ions induce fluorescence quenching through static complex formation between the arsenic species and surface functional groups on the N-MQDs. This interaction is highly selective, driven by coordination chemistry rather than nonspecific adsorption, providing a reliable basis for arsenic recognition. The subsequent addition of a competitive chelating ligand (*e.g.*, MBTZ) detaches the arsenic ions from the quantum dot surface, resulting in fluorescence recovery. Such reversibility demonstrates that the chemical surface states of MQDs can be tuned to allow multi-step logical sensing within a single portable device.

From an application perspective, the low detection limit of 30 nM for As^3+^ is well within regulatory standards for safe water monitoring. Importantly, the quantum dots were successfully integrated into a solid-state sensor suitable for direct wastewater analysis, showcasing practical portability. Beyond analytical performance, this study emphasizes the interplay between chemical surface modification (*via* nitrogen doping) and nanoscale coordination chemistry to achieve reversible, logic-based sensing. The approach highlights the potential for creating reusable or multi-analyte detection platforms, where the signal modulation is controlled entirely through chemical interactions on the quantum dot surface rather than additional instrumentation. This combination of photophysical robustness and chemical tunability provides a blueprint for developing advanced portable fluorescent sensors that are both environmentally compatible and operationally practical.

### 4.3. Molecularly imprinted electrochemical recognition of Tau protein for portable neurodiagnostics

Electrochemical detection of macromolecular biomarkers in portable settings demands precise chemical recognition and signal amplification strategies. A molecularly imprinted polymer (MIP) integrated with a Ti_3_C_2_T_*x*_ MXene, nitrogen-doped carbon dot, and ionic liquid nanocomposite provides a chemically and structurally optimized platform for Tau protein detection. The MXene scaffold offers high electrical conductivity and a large surface area, enabling efficient charge transport. Nitrogen-doped carbon dots contribute additional active sites and functional groups for noncovalent interactions, while the ionic liquid promotes ion mobility and reduces nonspecific fouling, collectively enhancing electrochemical performance.^77^

Selectivity arises from the molecular imprinting process, which creates cavities complementary in shape and functionality to the Tau protein. However, sensitivity is further amplified by synergistic chemical interactions within the nanocomposite. The interplay between MXene conductivity and carbon dot redox activity ensures rapid electron transfer and enhanced current response upon protein binding. This chemical engineering enables a linear detection range of 10–300 pg mL^−1^ with an impressively low detection limit of 1 pg mL^−1^, suitable for early-stage diagnostics and real-time monitoring of neurodegenerative disease progression.

The platform's screen-printed electrode configuration emphasizes portability, allowing miniaturized and reproducible deployment outside conventional laboratory settings. Validation in artificial serum demonstrated robust performance in complex matrices, highlighting practical translational potential. The study exemplifies how chemical design at the nanoscale can be integrated with macro-scale electrode architecture to create portable biosensors. Importantly, all functional enhancements are derived from engineered chemical interactions and material composition rather than modifications of the sensing principle itself, preserving intrinsic selectivity and robustness.

### 4.4. Dual-mode fluorescence–electrochemical detection of dopamine *via* Fe–N Co-doped MQDs

Fe–N co-doped MQDs illustrate the advantages of compositional tuning for multifunctional sensing. By introducing iron centers into N-MQDs, the material achieves simultaneous fluorescence and electrochemical detection of dopamine. Fluorescence quenching occurs through analyte-induced metal–ligand complexation, yielding a detection limit of 0.56 nM. In parallel, electrochemical oxidation of dopamine is catalyzed by Fe active sites within the MXene lattice, enhancing current response and allowing nanomolar-level detection across both cyclic voltammetry and differential pulse voltammetry.^78^

Integration onto both glassy carbon and screen-printed electrodes demonstrates adaptability for laboratory and portable sensor configurations. The extended linear range observed electrochemically supports quantification across physiologically relevant dopamine concentrations. Critically, the chemical nature of the Fe–N co-doping provides dual functionality: redox activity for electrochemical sensing and strong coordination for optical modulation, enabling reliable dual-mode detection. This redundancy enhances analytical confidence, mitigating false positives due to environmental perturbations common in portable settings. Mechanistically, the co-doping strategy demonstrates that chemical modification at the atomic level can transform a single-mode nanosensor into a multifunctional platform. The synergy between Fe coordination chemistry and MXene conductivity exemplifies how molecular-level design translates into macroscopic performance gains. Such dual-mode systems offer significant advantages for point-of-care neurochemical monitoring, where robustness, sensitivity, and operational reliability are essential.

**Fig. 6:**
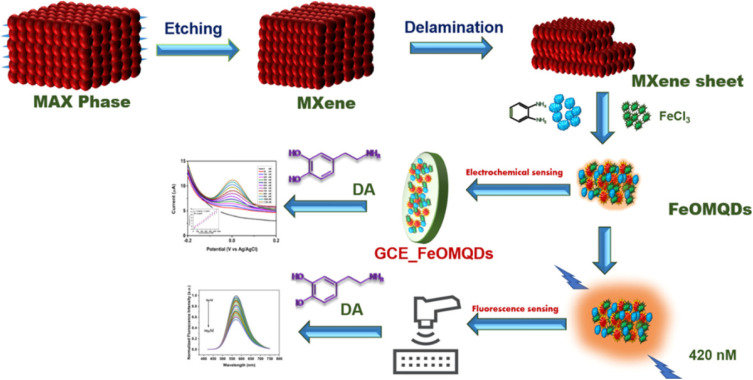
Schematic illustration of the synthesis of Fe–N co-doped MXene quantum dots (FeOMQDs) from MAX phase and their dual-mode fluorescence and electrochemical sensing of dopamine, highlighting photoluminescence quenching and electrocatalytic oxidation mechanisms. Adapted with permission from ref. 78. © 2026 Elsevier B.V.

The Fig. 6 schematically illustrates the synthesis and dual-mode sensing concept of Fe–N co-doped MXene quantum dots (FeOMQDs) for dopamine detection. Starting from the MAX phase, selective etching and delamination yield MXene sheets, which are subsequently transformed into FeOMQDs through a one-step hydrothermal process in the presence of FeCl_3_ and nitrogen-containing ligands. The resulting quantum dots inherit the high conductivity and rich surface chemistry of MXenes while incorporating iron coordination sites that endow multifunctional sensing capability. In the fluorescence pathway, dopamine interacts with surface Fe centers, forming metal–analyte complexes that induce efficient photoluminescence quenching, enabling sensitive optical detection at nanomolar concentrations. In parallel, immobilization of FeOMQDs on glassy carbon or screen-printed electrodes creates an electrochemically active interface, where synergistic interactions between Fe redox sites and the MXene matrix enhance dopamine oxidation currents. Together, the figure captures how atomic-level Fe–N co-doping converts MQDs into an integrated fluorescence–electrochemical platform, translating compositional design into robust, dual-mode analytical performance suitable for practical bioanalytical applications.

### 4.5. Smartphone-integrated dual-mode detection of norepinephrine using N-doped MQDs

The integration of N-MQDs with smartphone-based analytical readouts demonstrates a convergence of chemical design and consumer electronics for decentralized diagnostics. N-doped MXene QDs synthesized *via* microwave-assisted routes serve as dual probes for fluorescence and electrochemical detection of norepinephrine. Fluorescence modulation occurs through Förster resonance energy transfer, resulting in quenching and concentration-dependent color changes detectable *via* smartphone RGB analysis. This allows translation of nanoscale chemical events into quantifiable digital outputs.

Electrochemical detection complements the optical readout, leveraging the redox activity of norepinephrine and MQD surface functionalities. The platform achieves a detection range of 0.1–500 µM and low nanomolar limits in human serum, indicating both chemical sensitivity and robustness. Signal correlation between fluorescence and electrochemical readouts enhances analytical reliability, particularly in point-of-care environments where sample handling and environmental variability can affect performance.^79^

From a chemical perspective, nitrogen doping plays a dual role: enhancing photoluminescence and facilitating reversible surface interactions with norepinephrine. Coupled with smartphone-based digital analysis, this chemically engineered nanosensor enables rapid, user-friendly, and reproducible detection. The study highlights how deliberate atomic-level chemical modifications can translate into macroscale operational advantages in real-world portable diagnostic systems.

### 4.6. Smart paper-based platforms with N,B Co-doped MQDs for environmental chromium monitoring

Smart paper-based platforms utilizing nitrogen and boron co-doped MQDs combine chemical functionality with macroscopic practicality for environmental sensing. The MXene QDs are immobilized in polyethyleneimine-functionalized paper, enabling both selective detection and adsorption of dichromate ions (Cr_2_O_7_^2−^). Nitrogen and boron co-doping extends excitation wavelengths and stabilizes the quantum dots against oxidative degradation, enhancing operational reliability in environmental conditions.

Fluorescence quenching is rapid (∼10 s) with a quenching efficiency of ∼99.9%, while immersion and cyclic filtration modes achieve detection limits in the low nanomolar range. The high adsorption capacity (162.4 mg g^−1^) enables simultaneous capture and monitoring of Cr_2_O_7_^2−^, providing dual functionality that is particularly relevant for on-site water quality assessment. The chemical interactions underlying quenching involve coordination of dichromate ions with doped surface sites on the quantum dots, ensuring selectivity and reversible signal modulation.^80^

The study demonstrates how chemically engineered nanoscale materials can be integrated into scalable, low-cost, and portable macroscopic platforms. By coupling adsorption and sensing functions, the paper-based system exemplifies a practical solution for environmental monitoring, bridging nanoscale chemical design with field-deployable diagnostics. This approach reinforces the versatility of doped MQDs as chemically active cores within multifunctional, real-world sensing systems.

### 4.7. Multi-modal and multi-channel sensing strategies enabled by N-doped MQDs

Recent advances in sensing technologies highlight the increasing importance of multi-modal and multi-channel detection strategies to enhance analytical reliability, minimize false positives, and provide orthogonal verification within a single platform. In this domain, N-MQDs and their co-doped derivatives exhibit a unique combination of photophysical and electrochemical properties that can be synergistically harnessed for multi-modal sensing—a trend that aligns with the highest-impact Q1 biosensing literature.

At the core of multi-modal sensing is the integration of distinct transduction mechanisms (optical *vs.* electrochemical) within a unified material framework. This duality not only provides complementary measurement channels but also enables cross-validation of detected analytes—a particularly useful feature in point-of-care (POC) contexts characterized by environmental variability and user handling differences. For instance, Fe/N co-doped MQDs facilitate simultaneous fluorescence quenching and electrochemical oxidation of dopamine.^78^ The fluorescence channel operates *via* analyte-induced photoluminescence quenching through metal–analyte complexation, while the electrochemical channel capitalizes on the intrinsic electrocatalytic activity of Fe centers within the MXene matrix. Such orthogonal readouts inherently reduce the likelihood of false positives because a true positive event must satisfy both optical and electrochemical criteria.

Multi-modal design becomes even more compelling when combined with complementary material strategies. For example, smartphone-integrated platforms using N-MQDs exploit both fluorescence changes and electrochemical responses for norepinephrine detection.^79^ The optical channel serves as a rapid, visual, user-friendly indicator, while the electrochemical signal provides quantitative, high-precision confirmation. In decentralized healthcare scenarios, this layered sensing architecture increases confidence in results without requiring multiple instruments or specialist expertise. This aligns with recent advances in multimodal bioelectronics, where the integration of complementary signals is increasingly recommended for robust diagnostics.

Moreover, multi-channel strategies can combine adsorptive enrichment with sensing, as seen in smart paper platforms with N,B co-doped MQDs for dichromate monitoring.^80^ In these systems, initial adsorption at the interface concentrates the target analyte, amplifying the sensing signal upon detection. The dual functional role—capture and detection—effectively creates a sequential multi-step measurement channel that enhances analytical sensitivity and lowers limits of detection in real-world samples.

From a mechanistic perspective, multi-modal strategies exploit the atomic-level tunability of MQDs: heteroatom doping (N and co-dopants like Fe or B) regulates electronic structure, surface chemistry, and redox behavior—all determinants of both optical and electrochemical responses. This tunability distinguishes MQD-based systems from conventional nanomaterials, where orthogonal channels often require disparate materials that complicate integration. The ability to engineer a single nanomaterial with dual or multi-channel functionality facilitates miniaturization, reduces fabrication complexity, and enhances portability.

However, realizing true multi-modal platforms in practice presents challenges. Signal cross-talk between channels must be carefully mitigated, and calibration strategies need refinement to balance sensitivity and specificity across modalities. Additionally, integration into user-friendly interfaces (*e.g.*, smartphone-based) demands sophisticated data processing algorithms that can correlate and interpret signals from multiple channels synergistically. Nonetheless, the diversity of mechanisms demonstrated highlights a thriving design space where hierarchical signal encoding enables robust, context-aware sensing.^77,79^ As such, multi-modal design represents a forward-looking frontier in portable MQD-based sensors, with substantial potential to influence next-generation diagnostics.

### 4.8. Field stability, environmental robustness, and smart integration of MQDs platforms

Portable and point-of-care sensors face rigorous field conditions—mechanical stress, temperature fluctuations, humidity, and biofouling—that often degrade performance relative to controlled laboratory environments. Consequently, field stability and environmental robustness have emerged as core evaluation criteria in recent high-impact sensor research. N-MQDs and their derivatives demonstrate promising responses to these challenges due to their chemically versatile surfaces and ability to be embedded within mechanically resilient substrates.

A central aspect of field stability is mechanically robust integration of MQDs into sensor platforms. For example, solid-state arsenic sensors based on N-MQDs show reliable fluorescence response in wastewater environments, indicating resistance to shear and agitation often encountered in field sampling. Similarly, smart paper-based platforms with N,B co-doped MQDs leverage the structural support of functionalized paper matrices. These matrices provide mechanical integrity and restrict quantum dot mobility, preventing signal degradation due to detachment or redistribution under real-world handling. The high adsorption capacity in these systems simultaneously reinforces stability by anchoring analytes near sensing sites.

Environmental robustness also encompasses chemical resilience in the face of oxidative, pH, and redox stress. Co-doping strategies (*e.g.*, N,B or Fe,N) not only tailor sensing characteristics but also enhance the oxidative stability of MQDs, mitigating degradation pathways that can compromise long-term performance. For instance, the extended excitation wavelength and enhanced oxidation resistance observed in N,B co-doped platforms enable stable detection of dichromate ions across broad pH regimes—a common condition in environmental samples. This chemical robustness augments the operational lifetime of the sensor, reducing drift and maintaining calibration integrity during prolonged deployment.

Smart integration also extends to consumable electronics and user interfaces. Smartphone-integrated sensors using N-MQDs harness existing processing and display capabilities of mobile devices. Such integration not only democratizes access to analytical results but also supports field adaptability by leveraging ubiquitous hardware. Crucially, the interface design—whether RGB analysis for fluorescence or combined electrochemical readouts—must account for environmental noise, ambient light variation, and user-induced fluctuations. These considerations go beyond material design and require synergistic optimization of software, data normalization algorithms, and calibration models.

Moreover, portability demands rapid response and recovery characteristics. Smart paper platforms demonstrate <10 s response times for dichromate detection, underscoring the potential for real-time, *in situ* monitoring without prolonged equilibration. Rapid kinetics not only enhances user experience but also reduces cumulative environmental exposure risks during sampling. A similar need for rapid response drives designs in neurotransmitter and biomarker detection, where physiological variabilities and temporal fluctuations necessitate swift analytical feedback.

Addressing field robustness does introduce challenges. Strategies for biofouling resistance, long-term adherence to substrates, and preservation of nanoscale activity in complex matrices remain active research areas. Nonetheless, the collective evidence reveals that MQDs-based platforms exhibit a favorable balance of mechanical durability, chemical resilience, and smart integration potential.^76,80^ These attributes align with the expectations of high-impact sensor literature and lay the groundwork for future portable diagnostics capable of reliable performance in demanding real-world environments.

## 5. Challenges, emerging opportunities, and future directions

### 5.1. Challenges in portable N-MQDs platforms

Despite the remarkable advances in portable sensing enabled by N-MQDs, several technical challenges persist that limit widespread deployment. One key obstacle is signal reproducibility under real-world conditions. Variations in ambient light, temperature, humidity, and substrate handling can induce fluctuations in fluorescence intensity or electrochemical response, which can be particularly pronounced in miniaturized or paper-based platforms.^76^ Even minor heterogeneities in quantum dot distribution or surface functionalization can translate into significant deviations, underscoring the need for robust fabrication protocols.

Another critical challenge is stability and long-term functionality. N-MQDs, while chemically versatile, remain susceptible to oxidation, aggregation, or photobleaching over time, especially under high ionic strength or harsh environmental conditions.^78^ Co-doping strategies (*e.g.*, Fe/N or B/N) mitigate some of these effects, yet they introduce additional synthetic complexity and potential batch-to-batch variability. Additionally, interface compatibility between N-MQDs and flexible or smartphone-integrated substrates requires careful control of adhesion, mechanical resilience, and nanomaterial migration.

A third challenge is scalability and standardization. Current synthesis methods, such as hydrothermal or microwave-assisted approaches, are often optimized for laboratory-scale production.^79^ Translating these methods into reproducible, high-throughput manufacturing remains a non-trivial endeavor. Differences in doping concentration, particle size distribution, and surface passivation can lead to substantial sensor performance heterogeneity. Furthermore, standardization of testing conditions and calibration protocols across portable devices is essential to ensure reliability, especially for biomedical or environmental regulatory applications.

Finally, data interpretation and user accessibility present additional barriers. Multi-modal signals, while enhancing analytical confidence, require sophisticated algorithms for signal correlation and artifact rejection.^38,44^ Ensuring that point-of-care or field operators can reliably acquire and interpret data without specialist training is a non-trivial design constraint. Addressing these challenges is critical to transition N-MQDs from laboratory prototypes to robust, deployable platforms.

**Fig. 7:**
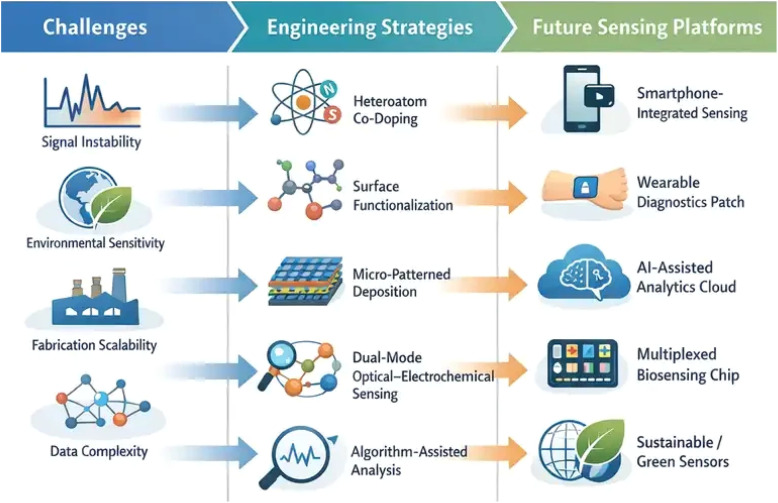
Conceptual roadmap illustrating the relationship between current challenges, engineering strategies, and future sensing platforms in portable N-MQDs systems, highlighting pathways toward smartphone-integrated, AI-assisted, and sustainable sensing technologies.

To provide a clearer conceptual perspective on the evolution of portable N-MQDs sensing systems, Fig. 7 summarizes the key relationships between current technological challenges, emerging material engineering strategies, and prospective sensing platforms. The schematic illustrates how fundamental limitations such as signal instability, environmental sensitivity, fabrication variability, and complex data interpretation motivate the development of advanced material and device engineering approaches. These include heteroatom co-doping, surface functionalization, micro-patterned deposition, and dual-mode sensing architectures. Through these strategies, N-MQD platforms are expected to progress toward next-generation sensing technologies characterized by smartphone-integrated detection, wearable diagnostics, AI-assisted signal processing, multiplexed analysis, and environmentally sustainable sensor designs.

### 5.2. Emerging opportunities for advanced sensing applications

The unique chemical and physical properties of N-MQDs provide fertile ground for next-generation sensing innovations. A notable opportunity lies in dual-mode or multi-modal detection, where single quantum dot architectures simultaneously deliver optical and electrochemical readouts.^36,50^ Such strategies enhance analytical fidelity, reduce false positives, and open pathways for multiplexed biomarker or pollutant detection. The tunable electronic structure provided by heteroatom doping allows targeted modulation of fluorescence wavelength, redox potential, and surface reactivity, making these platforms adaptable for diverse sensing scenarios.

Integration with smart, wearable, and mobile interfaces presents another promising avenue. Smartphone-assisted readouts^79^ and paper-based adsorption-detection systems^80^ exemplify the convergence of nanoscale functional materials with consumer electronics, enabling real-time data acquisition, storage, and transmission. This intersection of chemistry, materials science, and information technology represents a high-impact trend, particularly in decentralized diagnostics, environmental monitoring, and rapid-response field analysis.

Furthermore, N-MQDs can enable environmentally responsive or logic-gated sensing, leveraging “on/off/on” or multi-step recognition mechanisms.^38,41^ Such platforms can execute conditional detection sequences, allowing selective monitoring of analytes in complex matrices while simultaneously reducing interference. Combining this capability with adsorption-based enrichment^80^ amplifies analytical sensitivity, making them suitable for trace-level detection in water, biofluids, or other challenging media.

Lastly, the inherent versatility of MXene-based quantum dots supports tailored functionalization with biomolecules, polymers, or co-dopants to address highly specific analytical needs. This modularity facilitates the rapid prototyping of application-specific sensors while maintaining the core attributes of portability, sensitivity, and dual-mode operation.

### 5.3. Future directions in N-MQDs sensing

Looking ahead, several research directions are poised to define the next decade of N-MQDs-based sensing. First, scalable, reproducible synthesis remains essential. Transitioning laboratory-scale hydrothermal, microwave-assisted and chemical doping methods into standardized industrial processes will enable broader adoption and regulatory approval for biomedical or environmental applications.^39,44^

Second, intelligent platform integration is a key frontier. Coupling N-MQDs with flexible electronics, wearable patches, or autonomous microfluidic devices will require advanced interface engineering to maintain quantum dot activity while accommodating mechanical strain, fluid dynamics, and environmental perturbations. Integration with AI-assisted signal processing could further enhance data reliability, enabling context-aware interpretation and automated anomaly detection.

Third, the development of multi-analyte and logic-gated systems represents a significant opportunity. By engineering surface chemistry and quantum dot composition, sensors can simultaneously detect multiple biomarkers or pollutants with high selectivity, implementing complex decision-making algorithms at the material level. Coupling such capabilities with portable readout devices—smartphones, miniaturized potentiostats, or optical microarrays—can transform decentralized diagnostics.

Finally, addressing sustainability and environmental impact will likely become increasingly relevant. Designing N-MQD platforms using green synthesis routes, biodegradable substrates, and energy-efficient operation can enhance the societal impact and acceptability of these technologies. Collectively, these directions promise to elevate N-MQDs from innovative lab-scale materials to broadly deployable, high-performance sensing platforms in both healthcare and environmental monitoring contexts.

The Table 2 organizes the major challenges, emerging opportunities, and future research directions for portable N-MQDs platforms into a coherent framework. Each row highlights a specific platform aspect—ranging from signal reproducibility, environmental robustness, and fabrication scalability to multi-modal sensing, smartphone integration, and sustainability—while columns provide the underlying cause, current mitigation strategies, potential applications, and translational outlook. This format allows readers to directly link material design and system engineering with real-world analytical performance, emphasizing how chemical tuning, heteroatom co-doping, and interface optimization impact robustness, sensitivity, and usability in point-of-care and field-deployable contexts. By synthesizing complex technical and strategic information into a single reference, the table provides a clear, high-impact overview that supports both critical evaluation of existing platforms and the planning of next-generation N-MQD sensing systems.

**Table 2:** Engineering challenges and innovative strategies in portable N-MQDs platforms

Category	Critical aspect	Advanced strategy/Design approach
Signal reliability	Fluorescence and electrochemical fluctuation under variable ambient conditions	Micro-patterned deposition, surface energy tuning, and kinetic confinement of quantum dots
Photochemical & chemical stability	Oxidation, photobleaching, or aggregation in diverse media	Heteroatom co-doping (Fe/N, B/N), protective polymeric overcoats, and environmental encapsulation
Fabrication scalability	Consistency across batches in high-throughput production	Continuous-flow hydrothermal/microwave synthesis, automated quality control, and standardized particle characterization
Substrate adaptation	Integration with flexible, paper-based, or smartphone-linked platforms	Mechanical reinforcement, fiber-network entrapment, and strain-accommodating interface engineering
Data complexity management	Multi-modal or logic-gated signal interpretation	Algorithmic cross-validation, dual-mode correlation, and AI-assisted artifact suppression
Target selectivity	Multiplexed detection in complex matrices	Hierarchical surface functionalization, molecular imprinting, and multi-step recognition sequences
Dual-mode signal optimization	Balancing optical and electrochemical outputs	Controlled deposition thickness, conductive nanocomposite scaffolds, and wavelength/potential tuning *via* doping
Environmental sensing performance	Trace-level detection of ions or pollutants	Smart paper-based adsorption-detection, rapid quenching kinetics, and *in situ* enrichment strategies
User-centered deployment	Field-ready or point-of-care accessibility	Smartphone-based optical readout, portable potentiostats, and intuitive digital interfaces
Sustainability & future readiness	Eco-efficient design and operational sustainability	Biodegradable substrates, energy-efficient synthesis, modular sensor architectures

## 6. Conclusion

N-MQDs have emerged as a highly versatile class of nanomaterials, uniquely bridging the gap between chemical functionality and portable sensing platforms. Across diverse applications, from toxic metal ion detection and neurotransmitter monitoring to neurodegenerative biomarker quantification and environmental contaminant surveillance, these materials demonstrate remarkable optical and electrochemical responsiveness, tunable surface chemistry, and compatibility with miniaturized device architectures. Their intrinsic advantages, including long-wavelength emission, dual-mode transduction capability, and facile integration with flexible or smartphone-assisted platforms, collectively enable real-time, on-site analysis with high sensitivity and selectivity.

The review highlights how heteroatom doping, compositional modulation, and nanoscale engineering can systematically enhance analytical performance. For instance, dual-mode or multi-modal sensing strategies offer internal validation and redundancy, reducing false positives and improving robustness under variable environmental or biological conditions. Similarly, the development of smart paper-based or microfluidic-integrated formats demonstrates the potential for simultaneous detection and remediation, underscoring the functional versatility of N-MQDs beyond conventional sensing paradigms. Despite these advances, challenges remain in reproducibility, environmental stability, and scalable synthesis, which must be addressed to realize widespread deployment. Nevertheless, emerging trends—including logic-gated detection, AI-assisted readouts, and modular platform design—point to a future where N-MQDs underpin highly adaptable, field-deployable analytical systems. Collectively, the accumulated evidence confirms that N-MQDs are not only a platform for next-generation sensing but also a paradigm for integrating chemical functionality, nanostructured design, and user-oriented portability.

## Conflicts of interest

The authors declare that they have no known competing financial interests or personal relationships that could have appeared to influence the work reported in this paper.

## Data Availability

This article is a review and does not include any new experimental data. All data discussed and analyzed are derived from previously published studies, which are appropriately cited in the manuscript.

## References

[cit1] Gangadi J. R., Swain K., Pattnaik S. (2025). Emerging Trends and Future Perspectives in Bioanalysis. Bioanalytical Techniques.

[cit2] Kalambate P. K., Kumar V. (2025). Decentralized electrochemical biosensors for biomedical applications: From lab to home. Next Nanotechnol..

[cit3] Noorizadeh H. (2025). A review on the role of quantum dots in targeted drug delivery: Advances, functionalization, and applications in nanomedicine. Chem. Pharm. Lett..

[cit4] Jeon J., Choi H., Han G. R., Ghosh R., Palanisamy B., Di Carlo D., Ozcan A., Park S. (2025). Based Vertical Flow Assays for *in Vitro* Diagnostics and Environmental Monitoring. ACS Sens..

[cit5] Abushuhel M., Kumar A., Al-Hussainy A. F., Mohammed S., Panigrahi R., Noorizadeh H. (2025). Bromide perovskite quantum dot fluorescent sensors for food safety: Advances in pesticide and mycotoxin detection. J. Agric. Food Res..

[cit6] Qiu Y., Qiu Y., Zhou W., Lu D., Wang H., Li B., Liu B., Wang W. (2025). Advancements in functional tetrahedral DNA nanostructures for multi-biomarker biosensing: Applications in disease diagnosis, food safety, and environmental monitoring. Mater. Today Bio.

[cit7] Xu S., Qiao J., Chen Y., Wei J., Zhang W., Xu G., Wejrzanowski T., He W., Sun Z. (2025). (101) Facet growth directed by MXene-based quantum dots: A pathway to high-performance zinc-ion batteries. J. Energy Storage.

[cit8] Das P., Biswal L., Parida K. (2025). A review on MXene modified quantum dot photocatalysts for sustainable energy generation and environmental remediation. Catal. Sci. Technol..

[cit9] Xu S., Zhang X., Zheng T., Zhao Z., Shang C., Hu Z., Dong M., Qiao Y., Bai C., Zhang X., Sun G. (2025). Advancements in high-performance MXene composite fibers integrated with various functional materials: Fabrication, functionalization, property enhancement, and applications. J. Mater. Sci. Technol..

[cit10] Solangi N. H., Lingamdinne L. P., Karri R. R., Mubarak N. M., Mazari S. A., Koduru J. R. (2025). Emerging 2D MXene quantum dots for catalytic conversion of CO2. Carbon.

[cit11] Kumar R., Sahu A., Aadil K. R., Mishra Y. K., Kaushik A. (2025). Recent Progress in Multifunctional MXene Quantum Dots for Cancer Therapy. Curr. Opin. Biomed. Eng..

[cit12] Nie J., Zhang X., Wang M., Ou Y., Li S., Zhong P., Wang W., Zhu G., Ma X. (2025). MXene quantum dots decorated g-C3N4/BiOI heterojunction photocatalyst for efficient NO deep oxidation and CO2 reduction. Sep. Purif. Technol..

[cit13] Singh A., Mahapatra S., Prasad R., Singh S. K., Chandra P. (2025). Optoelectronic MXene quantum dots: frontiers in sensor technology for next-generation diagnostics and environmental monitoring. Nanoscale.

[cit14] Govindaraju R., Kim J. (2025). MXene-enabled fluorescent sensing systems: recent advances in biomolecule detection. Microchem. J..

[cit15] Sariga B. A. M., Kumar S., Rajeev R., Thadathil D. A., Varghese A. (2023). New horizons in the synthesis, properties, and applications of MXene quantum dots. Adv. Mater. Interfaces.

[cit16] Deng H., Hui Y., Zhang C., Zhou Q., Li Q., Du H., Hao D., Yang G., Wang Q. (2024). MXene− derived quantum dots based photocatalysts: Synthesis, application, prospects, and challenges. Chin. Chem. Lett..

[cit17] Liu W., Luo D., Zhang M., Chen J., Li M., Chen A., Xi S., Yu A. (2024). Engineered MXene quantum dots for micro-supercapacitors with excellent capacitive behaviors. Nano Energy.

[cit18] Yuan Y., Jiang L., Li X., Zuo P., Zhang X., Lian Y., Ma Y., Liang M., Zhao Y., Qu L. (2022). Ultrafast shaped laser induced synthesis of MXene quantum dots/graphene for transparent supercapacitors. Adv. Mater..

[cit19] Singh R., Kumar S., Bera S., Bhunia S. K. (2023). Trends in using fluorescent MXene quantum dots for selective detection and bioimaging applications: a review. ACS Appl. Nano Mater..

[cit20] Sun J., Du H., Chen Z., Wang L., Shen G. (2022). MXene quantum dot within natural 3D watermelon peel matrix for biocompatible flexible sensing platform. Nano Res..

[cit21] You H. R., Lee S., Lee D. H., Murali G., Nissimagoudar A. S., Kim Y., Park S., Lee J., Kim S. J., Park J. Y., Moon B. J. (2023). Organic solvent dispersible MXene integrated colloidal quantum dot photovoltaics. Adv. Energy Mater..

[cit22] Liu Y., Zhang W., Zheng W. (2022). Quantum dots compete at the acme of MXene family for the optimal catalysis. Nano-Micro Lett..

[cit23] Mousavi S. M., Hashemi S. A., Kalashgrani M. Y., Rahmanian V., Gholami A., Chiang W. H., Lai C. W. (2022). Biomedical applications of an ultra-sensitive surface plasmon resonance biosensor based on smart MXene quantum dots (SMQDs). Biosensors.

[cit24] Wan M., Jimu A., Yang H., Zhou J., Dai X., Zheng Y., Ou J., Yang Y., Liu J., Wang L. (2023). MXene quantum dots enhanced 3D-printed electrochemical sensor for the highly sensitive detection of dopamine. Microchem. J..

[cit25] Sharipov M., Uzokboev S., Nghia N. N., Azizov S., Ryu W., Tawfik S. M., Lee Y. I. (2024). Recent progress in Arduino-and smartphone-based sensors for biochemical and environmental analysis. TrAC, Trends Anal. Chem..

[cit26] Upadhyay S., Kumar A., Srivastava M., Srivastava A., Dwivedi A., Singh R. K., Srivastava S. K. (2024). Recent advancements of smartphone-based sensing technology for diagnosis, food safety analysis, and environmental monitoring. Talanta.

[cit27] Li D., Zhuang P., Sun C. (2024). Unlocking the potential of perovskite-based nanomaterials for revolutionary smartphone-based sensor applications. J. Mater. Chem. C.

[cit28] Chen J., Shao J., Sun R., Zhang W., Huang Y., Zheng J., Chi Y. (2023). Anion exchanges of water-stable perovskite nanocrystals in the pure water phase and applications in detecting halide ions *via* a smartphone-based sensing platform. Anal. Chem..

[cit29] Lim R. R., Huang Q., Ambrosi A., Bonanni A. (2024). Portable Smartphone-Assisted Graphene Quantum Dots Sensing Platform for the Detection of Gut Microbial Metabolites. ACS Appl. Nano Mater..

[cit30] Yue J., Ding S., An Y., Chen F., Zhang Q. (2024). Quantum dots colorimetric sensing system
based on paper-based aptasensor coupled with smartphone-based device. Measurement.

[cit31] Tian H., Jiao L., Wang K., Zhao X., Cao F., Dong D. (2022). Exploring smartphone-based environmental sensors through applying perovskite quantum dots. Chem. Eng. J..

[cit32] Lin C., Song X., Ye W., Liu T., Rong M., Niu L. (2024). Recent progress in optical sensors based on MXenes quantum dots and MXenes nanosheets. J. Anal. Test..

[cit33] Huang D., Wu Y., Ai F., Zhou X., Zhu G. (2021). Fluorescent nitrogen-doped Ti3C2 MXene quantum dots as a unique “on-off-on” nanoprobe for chrominum (VI) and ascorbic acid based on inner filter effect. Sens. Actuators, B.

[cit34] Jiang D., Wei M., Du X., Qin M., Shan X., Chen Z. (2022). One-pot synthesis of ZnO quantum dots/N-doped Ti3C2 MXene: Tunable nitrogen-doping properties and efficient electrochemiluminescence sensing. Chem. Eng. J..

[cit35] Wan M., Zhou J., Yang H., Dai X., Zheng Y., Xia Z., Wang L. (2022). Covalently N-doped MXene quantum dots for highly stable fluorescent Cu2+ ion sensor. ACS Appl. Nano Mater..

[cit36] Gou J., Zhao L., Li Y., Zhang J. (2021). Nitrogen-doped Ti2C MXene quantum dots as antioxidants. ACS Appl. Nano Mater..

[cit37] Zhang T., Dong C. S., Zhang L. Z. (2023). Solar-assisted-self-healing N-doped MXene quantum dots-based membrane with protein resistance for seawater desalination. Environ. Sci. Technol..

[cit38] Rajapriya G., Sangubotla R., Kim J. (2024). Synthesis of a fluorescent sensor by exploiting nitrogen-doped MXene quantum dots for the detection of dopamine. Korean J. Chem. Eng..

[cit39] Ding J., Tang C., Zhu G., Sun W., Du A., He F., Wu M., Zhang H. (2021). Integrating SnS2 quantum dots with nitrogen-doped Ti3C2T x MXene nanosheets for robust sodium storage performance. ACS Appl. Energy Mater..

[cit40] Kim S., Yang H., Jeong S., Lee T., Chae S., Lee J. H., Li O. L. (2023). Negative surface charge-mediated Fe Quantum dots with N-doped graphene/Ti3C2Tx MXene as chlorine-resistance electrocatalysts for high performance seawater-based Al-air batteries. J. Power Sources.

[cit41] Wei P., Chen Y., Zhou T., Wang Z., Zhang Y., Wang H., Yu H., Jia J., Zhang K., Peng C. (2022). Manipulation of Charge-Transfer Kinetics *via* Ti3C2 Tx (T=− O) Quantum Dot and N-Doped Carbon Dot Coloading on CdS for Photocatalytic Hydrogen Production. ACS Catal..

[cit42] Faraji M., Yousefzadeh S., Nassar M. F., Zahra M. M. (2022). MnCo2O4/N-doped graphene quantum dot vigorously coupled to MXene nanosheet: A bifunctional Oxygen electrocatalyst outperforms Pt/IrO2 benchmark electrocatalysts in metal-air batteries. J. Alloys Compd..

[cit43] Ding L., Zeng S., Zhang W., Guo C., Chen X., Peng B., Lv Z., Zhou H., Xu Q. (2022). Nitrogen-doped Ti3C2 MXene quantum dots/1D CdS nanorod heterostructure photocatalyst of highly efficient hydrogen evolution. ACS Appl. Energy Mater..

[cit44] Nguyen T. N., Doong R. A., Liu K. K. (2025). Nitrogen-doped Ti3C2 MXene-derived quantum dots for ultrasensitive detection of tetracycline in human serum. Microchem. J..

[cit45] Goda E. S., Jang W., Kim B. G., Wang D. H. (2025). Prussian blue analogue derived Al-doped CoN core-shell decorated by N-doped MXene
quantum dots for solid-state asymmetrical supercapacitors. J. Power Sources.

[cit46] Mohanty B., Giri L., Jena B. K. (2021). MXene-derived quantum dots for energy conversion and storage applications. Energy Fuels.

[cit47] Rajeev R., Babu A. M., Varghese A. (2025). Boron-/nitrogen-doped Ti 3 C 2 T x MXene quantum dot-based sensor for determining an acute kidney injury biomarker. RSC Adv..

[cit48] Zhou X., Zhang J., Huang D., Yi Y., Wu K., Zhu G. (2023). Nitrogen-doped Ti3C2 MXene quantum dots as an effective FRET ratio fluorometric probe for sensitive detection of Cu2+ and D-PA. Spectrochim. Acta, Part A.

[cit49] Kapuria A., Mondal T. K., Debnath A., Su Y. K., Saha S. K. (2025). Nitrogen, sulfur codoped MXene quantum dot–a superior HER electrocatalyst. Surf. Interfaces.

[cit50] Fatima S., Hakim M. W., Zheng X., Sun Y., Li Z., Han N., Li M., Wang Z., Han L., Wang L., Khan S. (2025). Constructing nitrogen-doped graphene quantum dots/tantalum carbide MXene heterojunctions as bifunctional catalysts for efficient water splitting. Int. J. Hydrogen Energy.

[cit51] Shao B., Liu Z., Zeng G., Wang H., Liang Q., He Q., Cheng M., Zhou C., Jiang L., Song B. (2020). Two-dimensional transition metal carbide and nitride (MXene) derived quantum dots (QDs): synthesis, properties, applications and prospects. J. Mater. Chem. A.

[cit52] Pu L., Zhang J., Jiresse N. K., Gao Y., Zhou H., Naik N., Gao P., Guo Z. (2022). N-doped MXene derived from chitosan for the highly effective electrochemical properties as supercapacitor. Adv. Compos. Hybrid Mater..

[cit53] Sun J., Shengping Zhang B. S., Alomar M., Alqarni A. S., Najla Alotaibi M. S., Badriah Alshahrani M. S., Alghamdi A. A., Kou Z., Shen W., Chen Y., Zhang J. (2023). Recent advances in the synthesis of MXene quantum dots. Chem. Rec..

[cit54] Sharbirin A. S., Akhtar S., Kim J. (2021). Light-emitting MXene quantum dots. Opto-Electron. Adv.

[cit55] Li D., Zheng X., Boutinaud P., Hu Y., Xiao S., Xu J., Wang C., Hou Y., He Z., Huang W., Kang F. (2024). An overview of nitrogen-doped MXenes and their recent developments. Responsive Mater..

[cit56] Ghosh S., Bera S., Kapuria A., Debnath A., Das P., Su Y. K., Saha S. K. (2025). Nitrogen and sulfur co-doped carbon quantum dot-decorated Ti 3 C 2 T x-MXenes as electrode materials for high-performance symmetric supercapacitors. Nanoscale.

[cit57] Govindaraju R., Paul J., Kim J. (2025). Electrochemical detection of neuroendocrine tumor biomarker normetanephrine using biocompatible FeN doped MXene quantum dot nanocomposites. Microchem. J..

[cit58] Cheng Y., Jiang B., Chaemchuen S., Verpoort F., Kou Z. (2023). Advances and challenges in designing MXene quantum dots for sensors. Carbon Neutralization.

[cit59] Safaei M., Shishehbore M. R. (2021). Energy conversion and optical applications of MXene quantum dots. J. Mater. Sci..

[cit60] Rajamanikandan R., Sasikumar K., Ju H. (2024). Ti3C2 MXene quantum dots as an efficient fluorescent probe for bioflavonoid quercetin quantification in food samples. Anal. Chim. Acta.

[cit61] Cai M., Wei X., Huang H., Yuan F., Li C., Xu S., Liang X., Zhou W., Guo J. (2023). Nitrogen-doped Ti3C2Tx MXene prepared by thermal decomposition of ammonium salts and its application in flexible quasi-solid-state supercapacitor. Chem. Eng. J..

[cit62] Lu C., Yang L., Yan B., Sun L., Zhang P., Zhang W., Sun Z. (2020). Nitrogen-doped Ti3C2 MXene: mechanism investigation and electrochemical analysis. Adv. Funct. Mater..

[cit63] Ren D., Cheng X., Chen Q., Xu G., Wei F., Yang J., Xu J., Wang L., Hu Q., Cen Y. (2023). MXene-derived Ti3C2 quantum dots-based ratiometric fluorescence probe for ascorbic acid and acid phosphatase determination. Microchem. J..

[cit64] Wang X., Zhang X., Cao H., Huang Y. (2022). An inorganic base stripping approach to synthesize N-doped Ti3C2 quantum dots as fluorescence nanoprobe for the simultaneous detection of Co2+ and Ag+ ions. Microchem. J..

[cit65] Liu M. Z., Li X. H., Yan H. T., Zhang R. Z., Cui H. L. (2023). Influence of N-doped concentration on the electronic properties and quantum capacitance of Hf2CO2 MXene. Vacuum.

[cit66] Alli U., McCarthy K., Baragau I. A., Power N. P., Morgan D. J., Dunn S., Killian S., Kennedy T., Kellici S. (2022). In-situ continuous hydrothermal synthesis of TiO2 nanoparticles on conductive N-doped MXene nanosheets for binder-free Li-ion battery anodes. Chem. Eng. J..

[cit67] Xia Z., Dai H., Chang J., Yang J., Wang H., Wang Y., Hui Z., Wang R., Sun G. (2023). Rheology Engineering for Dry-Spinning Robust N-Doped MXene Sediment Fibers toward Efficient Charge Storage. Small.

[cit68] Ahmed B., Tahir M. B., Ali A., Sagir M., Nassani A. A. (2025). First-principles study of the structural and electronic properties of N-doped Zr3C2 MXenes. Mater. Sci. Semicond. Process..

[cit69] Zhang F., Huang Z., Liu Y. Y., Qu Z. Y., Zhang Q. (2025). Novel active chlorine based-mechanism for 1O2-dominated tetracycline hydrochloride electrocatalytic oxidation over N-doped MXene. Sep. Purif. Technol..

[cit70] Cai L., Pan F., Zhu X., Dong Y., Shi Y., Xiang Z., Cheng J., Jiang H., Shi Z., Lu W. (2022). Etching engineering and electrostatic self-assembly of N-doped MXene/hollow Co-ZIF hybrids for high-performance microwave absorbers. Chem. Eng. J..

[cit71] Wei M., Du X., Zhang Y., Shan X., Wang W., Chen Y., Jiang D., Xu F., Shiigi H., Chen Z. (2022). Ultrasensitive self-driven photoelectrochemical aptasensor for lincomycin detection based on oxygen vacancy-tunable BiOBr nanosheet coupled with dual-function of N-doped Ti3C2 quantum dots. Biosens. Bioelectron.:X.

[cit72] Shi Y., Xiang Z., Cai L., Pan F., Dong Y., Zhu X., Cheng J., Jiang H., Lu W. (2022). Multi-interface assembled N-doped MXene/HCFG/AgNW films for wearable electromagnetic shielding devices with multimodal energy conversion and healthcare monitoring performances. ACS Nano.

[cit73] Tian W., Ren P., Hou X., Wang Y., Yuan S., Huang H., Jin Y. (2025). Advanced porous N-doped MXene/graphene/porous carbon as self-standing thick electrode for zinc-ion hybrid supercapacitors with wide working temperature range. Carbon.

[cit74] Zhang K., Zhao D., Qian Z., Gu X., Yang J., Qian Y. (2023). N-doped Ti3C2T x MXene sheet-coated SiO x to boost lithium storage for lithium-ion batteries. Sci. China Mater..

[cit75] Ahmed B., Tahir M. B., Ali A., Sagir M. (2025). Exploring the structural and electronic properties of N-doped Ti2C MXenes for novel applications in advanced materials and devices: A DFT study. Mater. Sci. Semicond. Process..

[cit76] Bera S., Bhunia S. K. (2025). Bright yellow fluorescent N-doped Ti 3 C 2 MXene quantum dots as an “on/off/on” nanoprobe for selective As 3+ ion detection. Nanoscale.

[cit77] Pakapongpan S., Poo-arporn Y., Poo-arporn R. P. (2025). Ti3C2Tx MXene nanosheets, N-doped carbon dots and ionic liquid nanocomposite as a molecular imprinted polymer based electrochemical sensor for tau protein. J. Mol. Liq..

[cit78] Govindaraju R., Paul J., Kim J., Kim J. (2026). Iron and nitrogen co-doped MXene quantum dots: A novel dual-mode electrochemical and fluorescence sensor for dopamine detection. J. Taiwan Inst. Chem. Eng..

[cit79] Chandran M., Chellasamy G., Veerapandian M., Dhanasekaran B., Govindaraju S., Yun K. (2025). Instant synthesis of nitrogen-doped Ti 3 C 2 MXene quantum dots for fluorescence and electrochemical dual-mode detection of norepinephrine with a portable smartphone assay. J. Mater. Chem. B.

[cit80] Yu Z., Deng C., Jiang S., Liu Y., Liu C., Seidi F., Zhang X., Huang Y., Wu W., Han J., Yong Q. (2025). Smart paper-based materials incorporating nitrogen and boron co-doped mxene quantum dots for rapid adsorption and sensitive detection of Cr2O72. J. Colloid Interface Sci..

